# Time to sense biofield (Prana) experiences between hands: A preliminary single blinded randomised controlled trial

**DOI:** 10.12688/f1000research.139737.1

**Published:** 2023-11-30

**Authors:** Vinu Vijayakumar, Srikanth Nagaraja Jois, Sumanth Mallikarjuna Majgi, Nagendra Prasad Krishnamurthy, Roopa Nanjunda swamy

**Affiliations:** 1 World Pranic Healing Foundation India Research Centre, Mysore, India; 2 Mysore Medical College and Research Institute, Mysore, India

**Keywords:** Cognition, complementary therapy, Pranic Healing, Yoga.

## Abstract

**Aims:** There is minimal research on the duration of biofield experiences. This preliminary study used the experiential learning practice of Master Choa Kok Sui’s hands sensitisation to determine the duration to experience biofield sensations in between hands and to find the relationship between learning style preferences and biofield sensations.

**Methods:** This randomised controlled, single blinded trial included 88 male and female pre-service teachers, aged 22.8 ±1.2 years. Learning Style questionnaire, Ruler drop test, and Six Letter Cancellation tests were administered to participants and randomised into two groups. The experimental (hands facing each other as introduced by Master Choa Kok Sui) and sham (hands facing opposite) groups practiced hands sensitisation. A semi-structured questionnaire was provided to gather information about biofield sensations and the time it took to experience these sensations between the hands.

**Results:** All (100%) of the participants in the experimental group (N=44) and 29.55% in the sham group (N=13) reported experiencing biofield sensations. A significant difference was found in, magnetic, temperature variation, and pain sensations between experimental and sham groups (X ^2^= 59.20, p<.001). In the experimental group, the average time taken to first experience magnetic sensation, other biofield sensations and temperature variation was 34.84±12.97, 40.28± 20.96 and 42.50±19.79, respectively. Minimum time taken to first experience biofield sensation was 5 seconds and lasted up to study duration of 120 seconds. In our observations, we found a significant negative correlation between the overall duration of experiences and the time it took for the first experience, while there was no correlation found between reaction time, attention, and the time needed to experience biofield sensations.

**Conclusions:** The duration to experience the novel biofield sensation was described in this study.

## Introduction

Complementary therapies are employed as a supplement to traditional healthcare systems around the world. They cover a various range of treatment methods, including biofield therapies. Healing touch, Pranic healing, Reiki, Qigong are examples of biofield therapies. According to the WHO Global Report on Traditional and Complementary Medicine, 12 member states have reported to use biofield therapies. ^1^ Biofield is variously known as “prana,” “ki,” and “chi,” among other names around the world. Researchers were able to demonstrate the modulating effect of the immune response on mice through biofield therapy. ^2^ Pranic Healing is suggested to promote subjective well-being by utilising ‘prana’ or vital energy. ^3^

Biofield sensations are the perception of subtle sensations without the presence of external stimuli. The sensations felt by the therapist during biofield therapy are helpful to measure and manage the client’s experience of biofield. ^4^^,^^5^ Energy sensations of hands were reported during Meditation on Twin Hearts Practice. ^6^ Hands sensitisation technique is used in pranic healing training. ^7^^,^^8^

The process of learning through experience is known as experiential learning, and it involves the construction of knowledge from real-life situations. Experiences from the environment influence the sensory process and perception of stimuli while engaging in experience-based activity or learning. ^9^ Based on the theory of experiential learning, learning styles have been represented by many theorists uniquely and combinations of factors have been expressed differently. ^10^ According to Honey and Mumford’s ^11^ model, learning styles consist of activist, reflector, theorist, and pragmatist. Activists are typically involved in and appreciate current experiences, reflectors are interested in analysing their experiences, theorists form conclusions based on their experiences, and pragmatists contemplate and take the next step. ^12^ Any stage of learning can be entered at any time, and the preferred learning style is influenced by attention, concentration, personality types and environmental factors. ^13^^,^^14^ Previously we have reported on the relationship between personality type and sensation between hands. ^15^ The time taken to perceive (reaction time) initially the subtle sensations between hands were not studied. Literature regarding the same is scarce.

Individual differences in biofield sensations are related to self-awareness and sensitivity to others. Experiences with extrasensory perception and an individual’s capacity to be aware of biofield collectively contribute to shaping biofield sensations. ^16^ Somatic sensations during biofield practice are connected to cognitive-perceptual and personality characteristics. ^17^^,^^18^ Identification and recognition of sensations varies from person to person based on expectations, motivations and the factors that truly influence and control that environment. ^19^ Reaction time influences the encoding of a natural activity through experiential learning. Learning by doing is much quicker in enacting a novel naturalistic activity compared to merely observing and then enacting the same activity performed by someone else. ^20^

Reaction time has been found to be related to the chronometry of cognitive processing, it helps in examining models of information processing and to explore the differences in cognitive abilities. ^21^ Chronometry is the science of measuring time. The time it takes for visual, auditory, and tactile sensations, ^22^^,^^23^ and the duration of olfactory and spontaneous sensations has been demonstrated in previous studies. ^24^ Focused attention to specific body parts are influential in detecting spontaneous sensations, the distribution of body fat and body mass index can impact sensory detection and pain sensitivity. For example, in comparison to individuals with a normal range body mass index, individuals with obesity have exhibited heightened sensitivity to pressure. ^23^^,^^25^ Chronometry of biofield sensations have not yet been studied. The connection of focussed attention, reaction time and body mass on biofield sensations during sensitisation of hands practice is also unknown. Here this investigation aimed to find the time taken to first experience the biofield sensations between hands and the entire duration of sensations experienced during hand sensitization, as well as any possible connections between these sensations and variables including reaction time, sustained attention, and BMI. The study also investigates the connection between these practitioners’ predominant learning styles and qualitative expressions.

## Methods

### Ethical considerations

The research was carried with the approval of the Independent Ethics Committee - World Pranic Healing Foundation, India (Ref: 2/2022/16/7/2022) on 20 ^th^ July, 2022, and this trial has been registered at Clinical Trial Registry of India (CTRI/2022/08/044540) on 1 ^st^ August, 2022. This study is reported inline with CONSORT guidelines. ^47^

### Study design

The study was conducted in August 2022, and it followed a randomised controlled participant blinded design. Participants of the study were relatively healthy pre-service teachers (students enrolled in Bachelor of Education) from Government College of Teacher Education (GCTE), Vasanth Mahal, Mysuru district. It is one of the teacher education centres in the Karnataka State under Department of State Educational Research and Training. Permission was obtained from Principal to conduct the experimental study among pre-service teachers. The sample size calculation was conducted in accordance with the guidelines set forth by Whitehead *et al*., ^26^ encompassing a 95% confidence interval, 80% power, and a standardized difference of 0.90, ^27^ while also accounting for a 20% dropout rate, resulting in 42 participants per arm.

This study was conducted at the college auditorium Hall. All participants gathered in the hall received information about the study. An informational sheet detailing the study was provided to each participant. Additionally, the study procedures were verbally explained to the participants, and this explanation was recorded to ensure accuracy and consistency in the process. Male and female candidates between 18 and 24 (22.8 ± 1.2) years were enrolled as per the inclusion criteria: students aged 18 or older, who agreed to take part in the study, and had no prior experience with Master Choa Kok Sui (MCKS) hands sensitisation practice were included.

Participants with known diabetes, hyperesthesia, peripheral neuropathy and sensory impairments) were excluded from the study. During baseline assessments, a qualified nurse inquired about participants’ health and whether they met the criteria to participate in the study. Additionally, medication taken in the previous 24 hours was recorded.

Pre-service teachers who signed a consent form and expressed an interest in participating in the study were accommodated in the hall. The demographic information, learning preferences and performance test results were obtained. The participants were divided into two groups (1:1) using the fishbowl method, and the groups were assigned using the coin toss method. The study participants were blinded to their condition. To prevent interference, only one group at a time engaged in sensitization practice within the hall, while the other group spent their time in a separate hall located 200 meters away from the study hall. The entire process of data collection and procedure administration was concluded over a span of five hours.

### Experimental group

Master Choa Kok Sui’s sensitization hand practise involved the steps as follows. ^7^ Practise abdominal breathing while sitting comfortably with the spine straight. Connect the tongue to the palate, press the centre of the palms with the thumb, place hands parallel to one another at three inches, move your hands slightly back and forth, and be aware of the centres of palms and the tips of fingers. The whole trial of Sensitization practice was conducted three times with one minute break (approximately) between each session consisting of 120 seconds.

### Sham group

**Figure 1.**
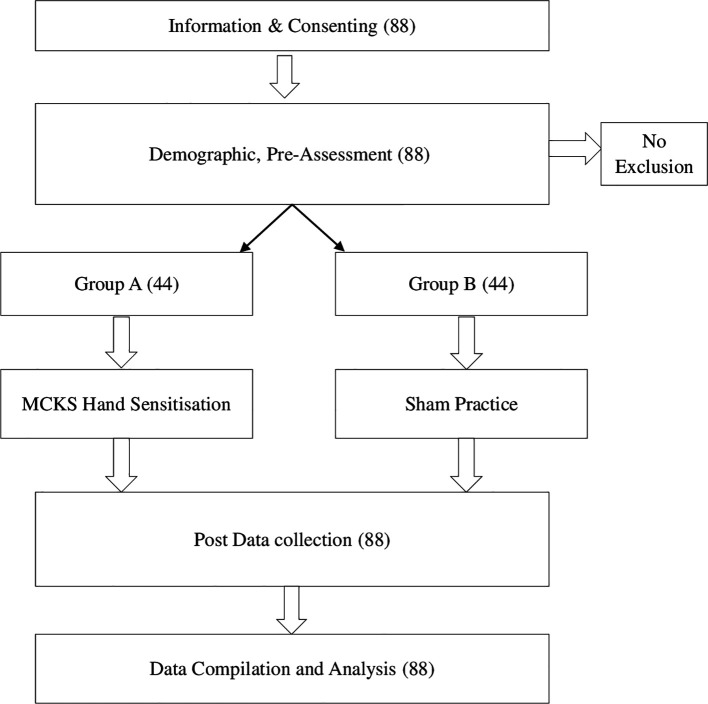
Flow chart.

The sham group got identical instructions to the experimental group, with the exception of palm facing in opposite directions and height variation between hands of approximately 3 inches ( Figure 1).

### Measurements

*Demographic details*

The participants’ age, gender, locality, medications taken within the last 24 hours, and highest educational qualification were recorded. Height was measured using a measuring tape, and weight in kilograms was divided by height in meters squared to calculate the BMI (Body Mass Index) using the formula: BMI = weight (kg) /height ^2^ (m ^2^).

Self-administered questionnaires (Learning Style, Sensation of Hands timing Questionnaire) and performance tests (Six letter Cancellation Test, Ruler Drop Test) were used to measure different outcome variables.

*Honey and Mumford:* Learning Styles Questionnaire: The Learning Styles Questionnaire (LSQ) is designed to measure learning preferences in individuals aged 16+, derived from the same conceptual basis as Kolb’s Learning Style Inventory. The Learning Style Questionnaire ^10^ contains 80 statements representing four learning styles that may be predominant in an individual, the activist, the reflector, the theorist, and the pragmatist. Construct validity to Kolb’s learning styles was confirmed using factor analysis. ^28^

*Six Letter Cancellation Test:* A sheet of randomly printed letters of alphabet was provided with 6 target letters. Subjects can either select a single target letter at a time or all six letters at once, and can follow random, vertical, or horizontal paths on cancelling the letters. They can cancel maximum possible letters within 90 seconds of time. ^29^

*Reaction Time:* To measure the Reaction time, Ruler Drop Test (RDT) was used Participants were asked to sit comfortably on a chair, ensuring that their dominant hand was positioned with the elbow bent at a 90-degree angle. The arm was then supported on the flat surface of the chair, with the open hand resting at the edge of the surface. A 60-cm-long ruler was dropped unexpectedly between the thumb and forefingers of their hand. They were instructed to quickly grasp the ruler as swiftly as possible. This ruler drop test (RDT) was repeated three times using the participant’s dominant hand. A rest interval of approximately 30 seconds was provided for each participant between each instance of dropping the ruler. The RT conversion was performed using the formula for a body in free fall under the influence of gravity (d= ½ gt ^2^). The test score is the distance reached, with a lower distance indicating better performance. ^30^

*The sensation of Hands Timing Questionnaire (SHTQ):* The Research Centre of the World Pranic Healing Foundation in India has created a new and self-administered questionnaire, which has undergone face validation. This questionnaire follows a semi-structured format and includes a combination of open-ended and closed-ended questions. These questions aim to capture information about participants’ experiences of sensations between their hands, the time it took to perceive these sensations, and how long the sensations persisted. Intensity of experiences among hands (left, right, both) and three open-ended questions on experience between hands were involved.

The open-ended questions included were 1) How did you feel about hand sensitisation practice? 2) In your opinion, what is the reason for experiencing these sensations between your hands 3) Describe the impact of the hand sensitisation experience on you?

*Experience of sensation of hands:* To keep track of how long first experience took and how long they lasted, a stopwatch was displayed in a projector. The duration between hands that the biofield experienced throughout a 120-second period was self-reported. Experiments were repeated three times and average time has been considered. The pattern of experiences between the hands and which hand is more prominent in feeling the sensation were explored.

### Data analysis

The experiences reported by participants in each group, along with the timings recorded in the Sensation Hands Timing Questionnaire, were thematically analysed using an inductive approach that aligned with the experiences during MCKS hands sensitisation. ^8^ Microsoft Word was used. The meaningful words containing experiences between the hands were initially grouped by the first author. The inclusion of experiences similar to biofield sensations was then categorized by both the first and second authors. Additionally, other authors of this paper verified the data, and queries were addressed. Subject’s sensation between hands during three attempts was derived for how long it typically takes to have first experience and how long each experience lasts. The average value of sensations was calculated. Participants of time taken to first experience the sensations were categorised into no experience, 1-10 sec, 10.1-20 sec, 20.1-30 sec, 30.1 to 40 sec, and more than 40 sec. The duration of sensation experiences was categorised into no experience, 1-20 seconds, 20.1-40 sec, 40.1-60 sec, 60.1-80 sec, and more than 80 sec. The average time for each category of biofield sensations was also calculated.

Furthermore, sensation experiences were classified as single, two sensations and three or more felt at same time. The association between the reported sensations and learning styles, as well as the association between the number of, biofield sensations and learning styles were analysed. Descriptive and inferential statistics using SPSS version 21 and Microsoft Excel were used to analyse the results.

## Results

The study comprised 88 students who were informed about the study and willingly took part by signing the consent form, with all participants meeting the eligibility criteria. Table 1 shows the demographic details and pre-assessment of pre-service teachers who participated in the trial. ^47^ The experimental and sham groups each consisted of 44 participants. There were 65 women (72.7% in the experimental group and 75% in the sham group). 77.3% had a rural background and there was no significant difference found in participants’ gender (χ ^2^ = 0.059, p = .808) or location (χ ^2^ = 1.035, p = .309) between the experimental and sham groups. The experimental group’s body mass index (BMI) was 21.95 ± 6.38 and that of the Sham group was 20.41 ± 3.50. No significant difference was found between the experimental and sham groups in terms of BMI (χ ^2^ = 4.286, p = .232). 14.77% of pre-service teachers had post-graduate degrees whereas the rest 85.23% had earned their degrees prior to enrolling in the pre-service teacher training programme. Importantly, the study did not experience any losses or exclusions following randomization, and no dropouts were recorded. Based on learning style theory, among all pre-service teachers, very high scores were identified in Active 49 (55.68%), Theorist 48 (54.55%), Pragmatist 38 (43.1%), and Reflector learning styles 27 (30.68%). Learning style preferences were categorized into very strong, strong, moderate, and low. No significant differences were found between the experimental and sham group pre-service teachers on active (2.295, p = .513), reflective (2.180, p = .703), theorist (1.307, p = .860), and pragmatic (3.197, p = .525) learning styles.

**Table 1.** Demographic details and pre-assessments among pre-school teachers.

Variables		Experimental		Sham		Statistic	
	Categories	N	%	N	%	χ ^2^	p- value
*Gender*	Male	12	27.3	11	25	0.059	.808
	Female	32	72.7	33	75		
*Location*	Urban	12	27.3	8	18.2	1.035	.309
	Rural	32	72.7	36	81.8		
*BMI*	Under	14	31.8	14	31.8	4.286	.232
	Normal	20	45.5	23	52.3		
	Pre-obese	6	13.6	7	15.9		
	Obese	4	9.1	0	0%		
LSP *-Active*	Very Strong	24	54.5	25	56.8	2.295	.513
	Strong	10	22.7	8	18.2		
	Moderate	10	22.7	9	20.5		
	Low	0	0	2	4.5		
LSP *-Reflective*	Very Strong	14	31.8	13	29.5	2.180	.703
	Strong	26	59.1	26	59.1		
	Moderate	3	6.8	4	9.1		
	Low	1	2.3	0	0		
	Very Low	0	0	1	2.3		
LSP *-Theorist*	Very Strong	23	52.3	24	54.5	1.307	.860
	Strong	9	20.5	9	20.5		
	Moderate	8	18.2	6	13.6		
	Low	4	9.1	4	9.1		
	Very Low	0	0	1	2.3		
LSP *-Pragmatic*	Very Strong	16	36.4	22	50.0	3.197	.525
	Strong	13	29.5	13	29.5		
	Moderate	11	25.0	5	11.4		
	Low	3	6.8	3	6.8		
	Very Low	1	2.3	1	2.3		
*Performance Tests*		Mean ± S.D.		Mean ± S.D.		t	p-value
*SLCT*		53.03 ± 15.77		47.29 ± 13.38		-1.837	p = .070
*RT (Sec)*		0.21 ± 0.03		0.21 ± 0.03		-.104	p = .917

BMI - Body Mass Index, LSP - Learning Style Preferences, SLCT - Six Letter Cancellation Test Scores, RT - Reaction time.

Performance tests involved the Six Letter Cancellation Test (SLCT) and Reaction Time (RT) Test. No significant differences were found between the experimental group (M = 53.03 ± 15.77) and the sham group (M = 47.29 ± 13.38) in SLCT (t = -1.837, p = .070) and RT (t = -0.104, p = .917). The mean reaction time of the experimental group and sham group were 0.21 ± 0.0 and 0.21 ± 0.03, respectively Right hand dominance was reported in all participants except one.

**Figure 2.**
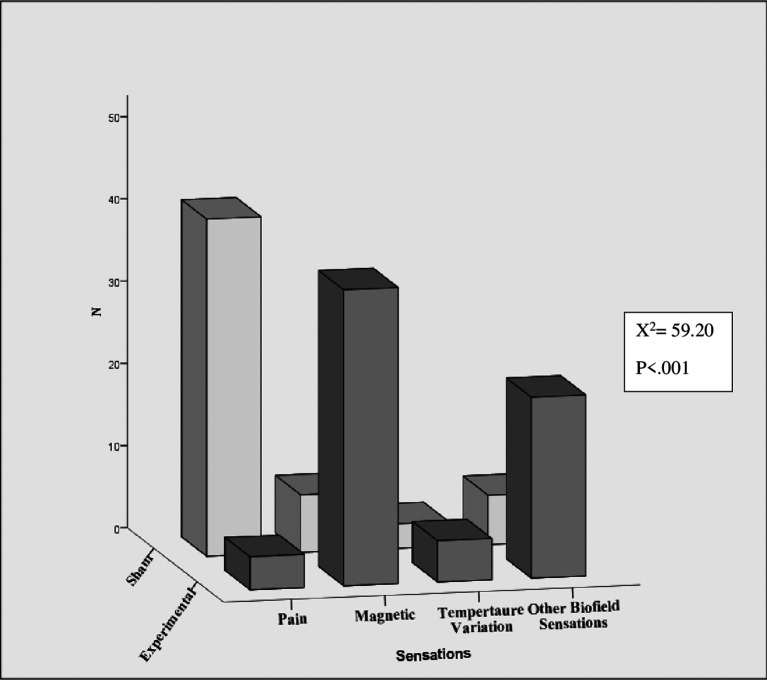
Sensations during hands sensitisation practice.

All the participants in the experimental group (N = 44) and 29.55% (N = 13) in the sham group reported experiencing biofield sensations. The experimental group reported magnetic sensations (81.81% (36/44) (“ *Magnetic pull between two palms,” “magnetic attraction“, “gravitational force”, and “Like a magnetic connection” etc*), 50% (22/44) reported other bio-field sensations *(“Felt hands are heavy”, “Something circling or spinning in mid of the palms”, “Felt like holding object”, “Something passing between hands”, “friction”, “vibration”, “lightness”, “heaviness”, “shaking”, “collision, pulsating” and “elastic force”* etc), 9.1% (4/44) reported pain experiences *(“Hand pain”, “shoulder pain” and “pain at the tip of tongue”*) and 11.4% (5/44) of them reported temperature variation *(“cold”, “heat” and “hotness”*) ( Figure 2).

In the sham group, 93.2% (41/44) reported pain experiences (“Hand pain”, “Left hand wrist pain”, “Little pain” and “Pain and attention”), 13.6% (6/44) reported other biofield sensation (“Felt a force like a thing between the hands”, “Hand pain with vibration”, “Weight lift feel”, “Hand pain and swimming feeling and Pulling” etc), 15.9% (7/44) reported magnetic sensations ( *“magnetic force*”, “ *Pain and magnetic*”) and 6.8% (3/44) of them reported awareness of temperature (“cold” and “pain with warmness”).

In terms of reporting magnetic sensations, pain, awareness of temperature, and other biofield sensation, there was a significant difference between the experimental and sham groups (χ ^2^ = 59.20, p < .001).

Table 2 shows there was no significant difference found between category of time taken to first experience the sensation between experimental and sham group participants.

**Table 2.** Time taken to experience the sensation during MCKS hand sensitisation.

Categories		Experimental	%	Sham	%	Statistics
Time taken to first experience	No sensation	0	0	1	2.3	χ ^2^ = 5.223, p = .389
	1-10 sec	0	0	2	4.5	
	10.1-20 sec	4	9.1	7	15.9	
	20.1-30 sec	10	22.7	7	15.9	
	30.1-40 sec	19	20.5	11	25	
	More than 40 sec	21	47.7	16	36.4	
Duration of experiences	No sensation	0	0	1	2.3	χ ^2^ = 1.167, p = .948
	1-20 sec	7	15.9	8	18.2	
	20.1-40 sec	10	22.7	9	20.5	
	40.1-60 sec	11	25	10	22.7	
	60.1-80 sec	12	27.3	12	27.3	
	More than 80 sec	4	9.1	4	9.1	

**Figure 3.**
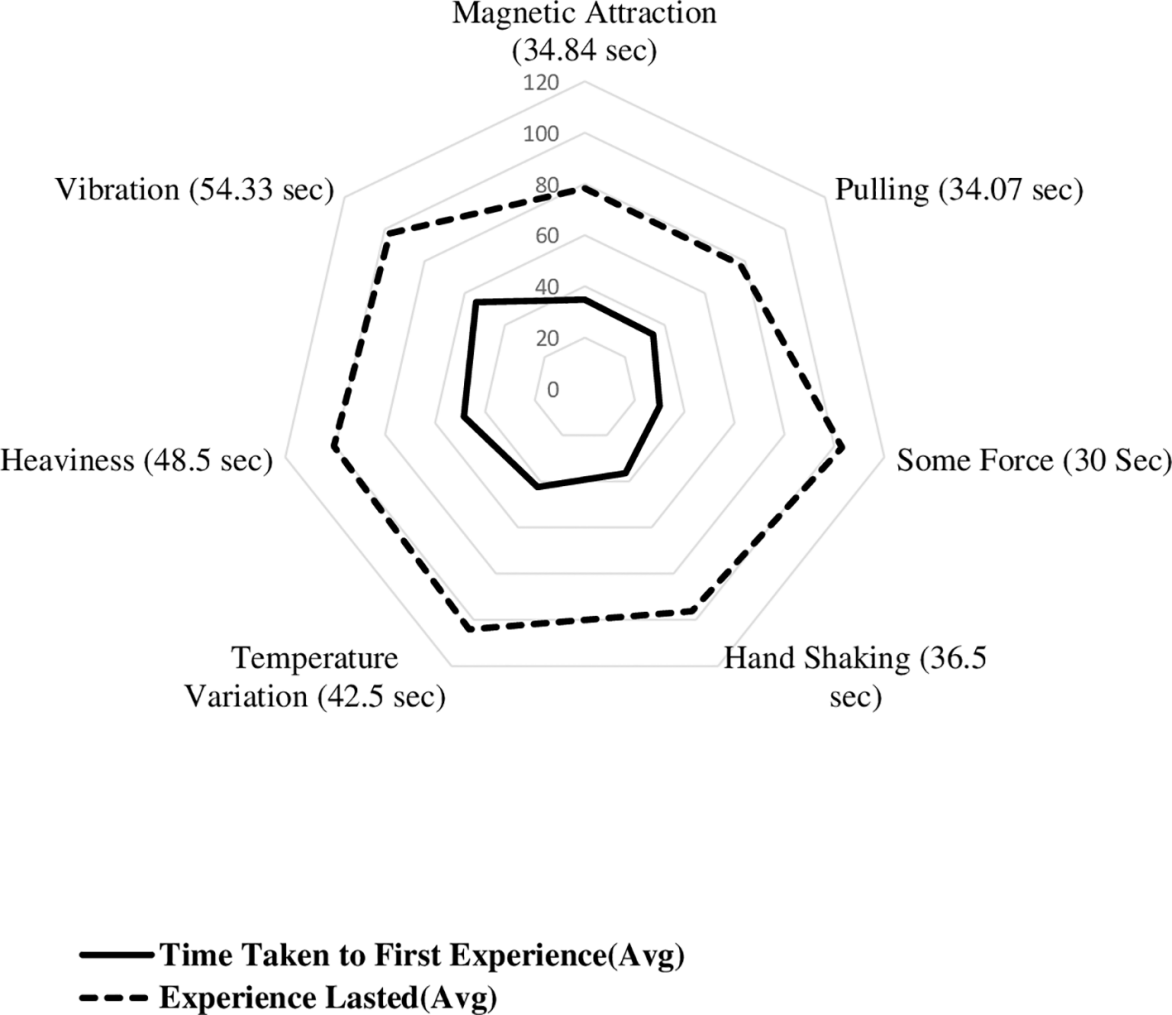
Mean time to experience different biofield sensations.

The categorised experiences and mean time taken to first experience the sensations and extend of time the sensations were sustained (Duration) were identified. Figure 3 displays the radial plot on mean time to experience different biofield sensations, including the time taken to first experience various sensations and the experiences lasted. The average time it took to first experience biofield sensations of magnetic attraction, pulling sensation, some force, handshaking experience, temperature variation, heaviness, and vibration were 34.84, 34.07, 30, 36.50, 42.5, 48.5, and 54.33 seconds, respectively. The time it took to first experience sensations of hand shaking, lightness, pulse beating, collision, and elastic force were 37, 40, 58, 58, and 61 seconds, respectively. The minimum time taken to first experience the sensation and maximum time taken to end it. The shortest time it took to first experience a sensation was 5 seconds, and the duration of sensations lasted up to 120 seconds. The minimum time taken to experience magnetic, pulling, some force, hand shaking, Temperature variation, heaviness and vibration sensations were 5, 20, 18, 22.5, 14, 32 and 26.67 seconds, respectively.

The mean duration of each sensation was calculated, with magnetic, pulling, some force, hand shaking, awareness of temperature, heaviness, and vibration having mean durations of 43.39 sec, 43.67 sec, 73 sec, 59.83 sec, 61.70 sec, 52.00 sec, and 43 sec, respectively. The duration of lightness, pulse beating, collision, and elastic force were reported as single values of 80 sec, 40 sec, 62 sec, and 61 sec, respectively. Based on the thematic categorisation, mean time taken to first experience the magnetic sensation (34.84 ± 12.97) sec, other biofield sensations is (40.28 ± 20.96) sec and temperature variations were also noted (42.50 ± 19.79).

Participants’ reported sensations between their hands were divided into three categories: only one type of sensations, two types of sensations, and three or more sensations felt at same time. More people (N = 25, 56.82%) reported having only one sort of sensation than had two types (N = 18, 40.91%), three or more sensations (N = 1, 2.27%). Table 3 shows the association of learning styles and number of combinations of experiences from SHTQ. Participants of reflective (χ ^2^ = 16.396, p = .012) and pragmatic (χ ^2^ = 16.653, p = .034) learning style had a significant association with sensation experiences between hands. 34.09% of Strong reflective and 20.45% very high pragmatic learning preferred were reported more single experiences than others.

**Table 3.** Association of biofield sensations and learning style preferences.

Variables		Sensations								Statistics
Learning Styles	Categories	Single	%	Two	%	Three or more	%	Total	%	χ ^2^ p -value
**Active**	*Very strong*	12	27.27	11	25	1	2.27	24	54.54	1.789, .775
	*Strong*	6	13.64	4	9.0	0	0	10	22.73	
	*Moderate*	7	15.91	3	6.82	0	0	10	22.73	
**Reflective**	*Very Strong*	8	18.18	6	13.64	0	0	14	31.82	16.396, .012
	*Strong*	15	34.09	11	25	0	0	26	59.09	
	*Moderate*	2	4.55	0	0	1	2.27	3	6.82	
	*Low*	0	0	1	2.27	0	0	1	2.27	
**Theoretic**	*Very Strong*	12	27.27	11	25	0	0	23	52.27	12.390, .054
	*Strong*	6	13.64	3	6.82	0	0	9	20.45	
	*Moderate*	6	13.64	2	4.55	0	0	8	18.18	
	*Low*	1	2.27	2	4.55	1	2.27	4	9.09	
**Pragmatic**	*Very Strong*	9	20.45	7	15.91	0	0	16	36.36	16.653, .034
	*Strong*	6	13.64	7	15.91	0	0	13	29.55	
	*Moderate*	7	15.91	4	9.09	0	0	11	25	
	*Low*	2	4.55	0	0	1	2.27	3	6.82	
	*Very Low*	1	2.27	0	0	0	0	1	2.27	

**Figure 4.**
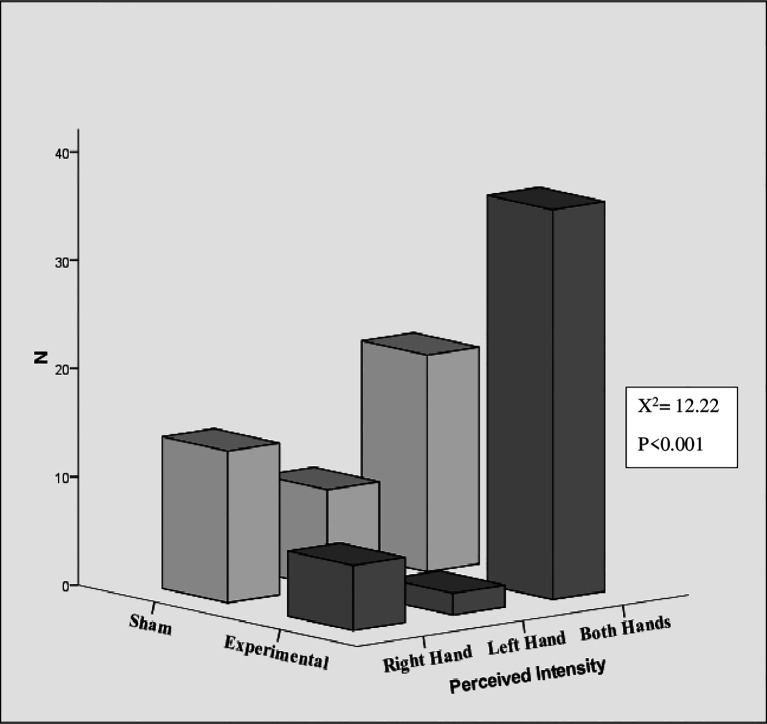
Perceived intensity of sensations in hands.

Figure 4 shows the perceived intensity of sensation in hands. A significant difference was found between the experimental and sham groups in the perceived intensity of sensation in the hands (left, right, and both) (χ ^2^ = 12.22, p < .001, N = 87). The intensity of experiences within the experimental group also were differed depending on whether they occurred in the left hand, right hand, or both hands (χ ^2^ = 47.09, p < .001, N = 44). In the experimental group, 81.81% (36) reported the intensity of sensation experiences in both hands. 13.6% (6/44) of participants reported experiencing intensity at right hand, while only 4.54% (2/44) reported experiencing sensations in their left hand. In the sham group, 35.7% of participants experienced the intensity of the sensation in both hands, while 20.9% and 32.6% experienced the intensity in their left and right hands, respectively. However, within groups there was no significant difference.

A significant negative correlation (r = -0.240, p = .025) exists between time taken to first experience sensation and duration of experiences among all participants. In the experimental group, the correlations of duration of experiences and time taken to first experience were negatively correlated (r = -0.326, p = .009,). No other correlations were found to be significant.

**Figure 5.**
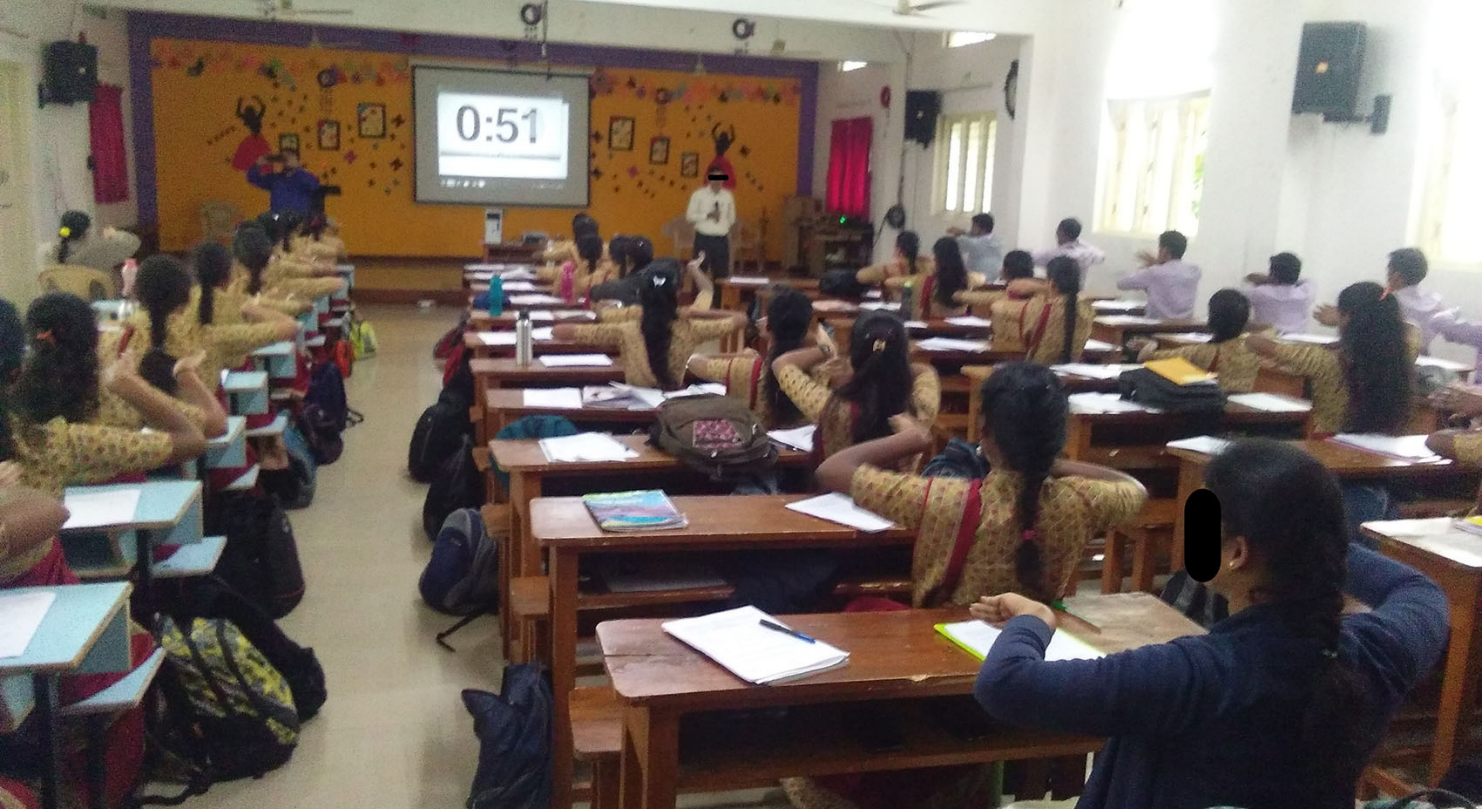
Experimental sessions of Sham group. All participants gave consent for their picture to be used.

In open ended questions, participants expressed that the experiences were magical, good, and novel sensations were felt during the practice. Improvement in respiration, reduction of stress, calmness and improvement in mood and concentration were common among experimental group participants as shown in Table 4. Participants reported their opinion about the reason for experiencing biofield sensations and the impact of hands sensitisation practice. The reason for experiencing the sensations by participants reported attention and concentration, blood circulation, sense of calm, coordination of sensory organs in the body, etc. Self-reported and sparsely described feedback was noticed in sham group ( Figure 5).

**Table 4.** Qualitative responses to open-ended questions.

Experimental group	Sham group
**In your opinion, what is the reason for experiencing these sensations between your hands?**	
*“May be because of attentively concentrated. There might be some inter relation between hands” - **E3***	“ *Nothing significant” - **P4***
*“Our body contains so many ionic particles, when we rub the hands and relax and moving, the ionic particle attracts other particles” - **E6***	“ *Right hand pain” - **P6***
*“Five sensory organs coordinate with one another like tongue touches upper nasal region and all concentration lay on movement of hand and respiration” - **E9***	“ *I do not feel anything” - **P24***
*“I think circulation of blood and movement of oxygen throughout the hands/body” - **E18***	“No experience *” - **P10***
*“According to me, my curiosity, attention and concentration and my involvement is the reason” - **E29***	*“Opposite direction and magnetic force” - **P32***
**Describe the impact of the hand sensitisation experience on you?**	
*“Freeness to hands and it helps to control respiration rate by ourselves” - **E9***	*“Improvement in the breathing and concentration” - **P34***
*“I think I feel cool mind. It is like a one type exercise for me. My stress and hands become free” - **E8***	*“It was a different experiment and would like to try it again” - **P28***
*“I feel, I am released of mental stress, my mind is fee and felt like a load of tension is removed from my mind” - **E16***	“ *Got awareness regarding hand movement and its reaction” - **P17***
*“I have a sense of calm. I have more anger and less patience. But from this experiment, I found that patience, calmness is a medicine for anger. Not having habit of meditation but it helped me” - **E37***	*“No impact on me - **P10***
*“If we do it daily, there will be no thoughts. Our mood will be good. It helps to learning” - **E30***	*“I felt hand power increased” - **P9***

## Discussion

This randomized controlled trial on sensitization between hands has demonstrated that both the experimental and sham groups self-reported an experience biofield sensations ( *prana*) in between their hands. Furthermore, it has been determined that the interpretation of these sensations based on time is a viable approach. A survey of 18 people found that they perceived spontaneous sensations more in their hands than feet. ^31^ In the current study, 57 participants experienced biofield sensation between their hands. The sham group participants experienced less (13) biofield sensations compared to experimental group (44). Majority of sham group participants reported hand, wrist, and arm pain during the experiment. It is possible that during the experience of pain, individuals may unconsciously direct their attention towards the affected area in the hands. ^32^ This may be one of the reasons for not experiencing biofield sensations. The possibility of not experiencing sensations exists owing to the procedure of hand sensitising and sensitivity of hand. Hence the data on the chronometry and biofield energy experiences of the experimental group were analysed further based on duration. The time taken to first experience the sensations of magnetic, other biofield and temperature variation are 34.84, 40.28 and 42.50, respectively.

People who frequently experience spontaneous sensations in their left hand, is due to the dominant right hemisphere activity of the brain. ^23^ In this investigation, biofield sensations were experienced in both the hands of participants; they reported the intensity of experiences in both the hands. It is possible that both hemispheres are activated during MCKS hands sensitisation. People who followed MCKS Hands sensitisation procedure felt different biofield sensations in between hands. It positively affected the physical and psychological wellbeing. ^33^ Biofield sensations have been reported following guidance or by following certain procedures mentioned in the biofield practices. ^34^

Somatic spontaneous sensations like tingling, temperature, beats, pulses, and pins have been previously investigated by detecting the spontaneous sensations within 10 seconds. Older participants reported more spontaneous sensations and duration of sensations when compared to youth population. ^35^ Magnetic field, self-awareness, sensitivity to others and conscious intent are the factors influencing biofield awareness. ^16^^,^^36^^–^^39^ So, the chances of variations in sensations are natural. MCKS sensitisation has been used as a learning practice to explore more about the subtle sensations or *pranic energy* sensations. ^7^ According to the findings, different participants experienced biofield sensations for varying lengths of time during hand sensitization practice.

Based on the context, environment, teaching method and subject of learning, the learning style preferences may get changed. ^40^ There was no association found between sensation between hands and learning styles in this study, but the learning styles were associated to number of experiences. According to the attention processing model, participants are able to pay attention to a specific experience due to the difficulty of observing all other sensations at the same time. ^41^ Self-analysis or introspection is the core to reflective learning. Complex Judgemental skills and problem-solving ability are the essential qualities of reflective thinkers. ^42^ Recording the intricate interplay of various sensations over time can be challenging for self-interpretation. Consequently, participants might have opted to focus on individual biofield experiences throughout the entire practice session. For instance, sensations like “Magnetic with Cold or Warm” or “Pulling along with Other Sensations,” or experiencing three or more sensations simultaneously, could have posed difficulties in accurately reporting their timings. Similarly, individuals who lean toward pragmatic approaches tend to simplify matters by highlighting single experiences when drawing conclusions. Hence, participants with higher reflective and pragmatic tendencies displayed a tendency to articulate individual sensations rather than complex combinations as their reported experiences.

The negative correlation between the time taken to have the first experience and the overall duration of experiences in the experimental group suggests a complex relationship influenced by factors such as learning, adaptation, and familiarity.The time taken to experience biofield sensation were unrelated to reaction time, sustained attention, or body mass index. One study discovered that, paying attention to the hands improves the duration of experiences. ^35^ Separating the conscious and unconscious sensory processes and connecting with attention is a difficult task. ^43^ Mind wandering awareness has been linked to perception of spontaneous stimulus. ^44^ One study revealed that the intensity of experience influences body mass index. ^45^ Participants can have a better understanding of biofield experiences by receiving further training and learning about biofield-related therapy. ^46^ In this study, hands-sensitization practise in the experimental group had a favourable effect on the participants, and the qualitative feedback supported the idea that biofield sensations improve current moods or states. ^33^

This procedure’s replicability offers potential for future biofield research, with follow-up studies involving diverse participant groups to validate observed results’ consistency on time taken to experience biofield sensations.

### Limitations of this study

The study’s questionnaire is unstandardized, necessitating the need to develop an instrument to measure time-related sensations between hands. Participants aged 18-24 were included, excluding older populations.

### Implications

This study findings may inspire biofield researchers to conduct time-based cognition and perception experiments. Based on MCKS hand sensitization, a theory on biofield sensations might be constructed, which could then be utilised as a tool or technique for studying numerous subtle sensations.

### Future direction

Further research on the duration of biofield sensations using chronometry is needed. Research is needed on specific biofield sensation-based approaches, as neuropsychological evidence on palm sensations is limited. It’s unclear if cortical brain activation is related to pranic energy experiences. Both spontaneous sensations and MCKS Hands Sensitisation experiences start without stimulus, but differ in methods. Investigation is needed to understand similarities and differences.

## Conclusion

This research emphasises the significance of recognising biofield sensations in hands sensitization practises. The MCKS hands sensitization practise research looked at biofield sensations between the hands under controlled settings. It kept track of time-related evaluations. It took 34.84, 54.33 and 42.5 seconds on average to initially feel magnetic attraction, vibration and temperature variation between hands. In the experimental group, there was a negative correlation between the time for the first experience and the overall duration of the experiences.

## Data Availability

Figshare: Time to experience Biofield Sensations Between Hands – A Randomised Control Trial, https://doi.org/10.6084/m9.figshare.24024948.v1. ^47^ This project contains the following underlying data:
- Consent and Demographic Details.pdf - Master Sheet_ Pre service Teachers.xlsx Figshare: CONSORT checklist for ‘Time to sense biofield (Prana) experiences between hands: A preliminary single blinded randomised controlled trial’, https://doi.org/10.6084/m9.figshare.24024948.v1. ^47^ Data are available under the terms of the Creative Commons Attribution 4.0 International license (CC-BY 4.0).
