# Time to sense biofield (Prana) experiences between hands: A preliminary single blinded randomized placebo controlled trial

**DOI:** 10.12688/f1000research.139737.3

**Published:** 2024-07-24

**Authors:** Vinu Vijayakumar, Srikanth Nagaraja Jois, Sumanth Mallikarjuna Majgi, Nagendra Prasad Krishnamurthy, Roopa Nanjunda swamy

**Affiliations:** 1 World Pranic Healing Foundation India Research Centre, Mysore, India; 2 Mysore Medical College and Research Institute, Mysore, India

**Keywords:** Cognition, complementary therapy, Pranic Healing, Yoga.

## Abstract

**Aims:**

There is minimal research on the duration of biofield experiences. This preliminary study used the experiential learning practice of Master Choa Kok Sui’s hands sensitisation to determine the duration to experience biofield sensations in between hands and to find the relationship between learning style preferences and biofield sensations.

**Methods:**

This randomized placebo controlled, single blinded trial included 88 male and female pre-service teachers, aged 22.8±1.2 years. Participants completed a ruler drop test for reaction time, and Six Letter Cancellation test for measuring attention, learning style questionnaire for preferred method of learning, before randomization. The experimental (hands facing each other as introduced by Master Choa Kok Sui) and sham (hands facing opposite) groups practiced hands sensitisation. A semi-structured questionnaire was provided to gather information about biofield sensations and the time it took to experience these sensations between the hands.

**Results:**

All (N=44) the participants in the experimental group and 13 participants in the sham group reported experiencing biofield sensations. A significant difference was noticed in experiencing magnetic (X ^2^ = 38.247, p ≤ .001), physical sensations of energy (X ^2^ = 12.02, p ≤ .001) and pain (X ^2^ = 62.259, p ≤ .001) among the experimental and sham group . In the experimental group, the average time taken to first experience magnetic sensation, other biofield sensations and temperature variation was 34.84±12.97seconds, 40.28±20.96 seconds and 42.50±19.79 seconds, respectively. Minimum time taken to first experience biofield sensation was 5 seconds and lasted up to study duration of 120 seconds. There was no correlation found between reaction time, sustained attention, and the time needed to experience biofield sensations.

**Conclusions:**

This study highlights importance of Master Choa Kok Sui hand sensitization in controlled setting revealing differences in experiences of various biofield sensations, showing valuable time-related insights and variability of sensation based on preferred learning.

## Introduction

Complementary therapies are employed as a supplement to traditional healthcare systems around the world. They cover a various range of treatment methods, including biofield therapies. Healing touch, Pranic healing, Reiki, Qigong are examples of biofield therapies. According to the WHO Global Report on Traditional and Complementary Medicine, 12 member states have reported to use biofield therapies. ^1^ Biofield is variously known as “prana,” “ki,” and “chi,” among other names around the world. Researchers were able to demonstrate the modulating effect of the immune response on mice through biofield therapy. ^2^ Pranic Healing is suggested to promote subjective well-being by utilising ‘prana’ or vital energy. ^3^

Biofield sensations are the perception of subtle sensations without the presence of external stimuli. The sensations felt by the therapist during biofield therapy are helpful to measure and manage the client’s experience of biofield. ^4^^,^^5^ Energy sensations of hands were reported during Meditation on Twin Hearts Practice. ^6^ Hands sensitisation technique is used in pranic healing training. ^7^^,^^8^

The process of learning through experience is known as experiential learning, and it involves the construction of knowledge from real-life situations. Experiences from the environment influence the sensory process and perception of stimuli while engaging in experience-based activity or learning. ^9^ Based on the theory of experiential learning, learning styles have been represented by many theorists uniquely and combinations of factors have been expressed differently. ^10^ According to Honey and Mumford’s ^11^ model, learning styles consist of activist, reflector, theorist, and pragmatist. Activists are typically involved in and appreciate current experiences, reflectors are interested in analysing their experiences, theorists form conclusions based on their experiences, and pragmatists contemplate and take the next step. ^12^ Any stage of learning can be entered at any time, and the preferred learning style is influenced by attention, concentration, personality types and environmental factors. ^13^^,^^14^ Previously we have reported on the relationship between personality type and sensation between hands. ^15^ The time taken to perceive (reaction time) initially the subtle sensations between hands were not studied. Literature regarding the same is scarce.

Individual differences in biofield sensations are related to self-awareness and sensitivity to others. Experiences with extrasensory perception and an individual’s capacity to be aware of biofield collectively contribute to shaping biofield sensations. ^16^ Somatic sensations during biofield practice are connected to cognitive-perceptual and personality characteristics. ^17^^,^^18^ Identification and recognition of sensations varies from person to person based on expectations, motivations and the factors that truly influence and control that environment. ^19^ Reaction time influences the encoding of a natural activity through experiential learning. Learning by doing is much quicker in enacting a novel naturalistic activity compared to merely observing and then enacting the same activity performed by someone else. ^20^

Reaction time has been found to be related to the chronometry of cognitive processing, it helps in examining models of information processing and to explore the differences in cognitive abilities. ^21^ Chronometry is the science of measuring time. The time it takes for visual, auditory, and tactile sensations, ^22^^,^^23^ and the duration of olfactory and spontaneous sensations has been demonstrated in previous studies. ^24^ Focused attention to specific body parts are influential in detecting spontaneous sensations, the distribution of body fat and body mass index can impact sensory detection and pain sensitivity. For example, in comparison to individuals with a normal range body mass index, individuals with obesity have exhibited heightened sensitivity to pressure. ^23^^,^^25^ Chronometry of biofield sensations have not yet been studied. The connection of focussed attention, reaction time and body mass on biofield sensations during sensitisation of hands practice is also unknown. Here this investigation aimed to find the time taken to first experience the biofield sensations between hands and the entire duration of sensations experienced during hand sensitization, as well as any possible connections between these sensations and variables including reaction time, sustained attention, and BMI. The study also investigates the connection between these practitioners’ predominant learning styles and qualitative expressions.

## Methods

### Ethical considerations

The research was carried with the approval of the Independent Ethics Committee - World Pranic Healing Foundation, India (Ref: 2/2022/16/7/2022) on 20 ^th^ July, 2022, and this trial has been registered at Clinical Trial Registry of India (CTRI/2022/08/044540) on 1 ^st^ August, 2022. This study is reported in line with CONSORT guidelines. ^47^

### Study design

The study was conducted in August 2022, and it followed a randomised controlled participant blinded design. Participants of the study were relatively healthy pre-service teachers (students enrolled in Bachelor of Education) from Government College of Teacher Education (GCTE), Vasanth Mahal, Mysuru district. It is one of the teacher education centres in the Karnataka State under Department of State Educational Research and Training. Permission was obtained from Principal to conduct the experimental study among pre-service teachers. The sample size calculation was conducted in accordance with the guidelines set forth by Whitehead *et al*., ^26^ encompassing a 95% confidence interval, 80% power, and a standardized difference of 0.90, ^27^ while also accounting for a 20% dropout rate, resulting in 42 participants per arm.

This study was conducted at the college auditorium Hall. All participants gathered in the hall received information about the study. An informational sheet detailing the study was provided to each participant. Additionally, the study procedures were verbally explained to the participants, and this explanation was recorded to ensure accuracy and consistency in the process. Male and female candidates between 18 and 24 (22.8 ± 1.2) years were enrolled as per the inclusion criteria: students aged 18 or older, who agreed to take part in the study, and had no prior experience with Master Choa Kok Sui (MCKS) hands sensitisation practice were included. Students above 18 are well-suited to analyse their learning style preferences due to their self-awareness and cognitive maturity. Understanding these preferences helps them tailor their study habits for better performance, efficiency, and engagement. It also fosters adaptability, making them versatile learners and benefiting them in future educational and professional contexts.

Participants with known diabetes, hyperesthesia, peripheral neuropathy and sensory impairments) were excluded from the study. During baseline assessments, a qualified nurse inquired about participants’ health and whether they met the criteria to participate in the study. Additionally, medication taken in the previous 24 hours was recorded.

**Figure 1.**
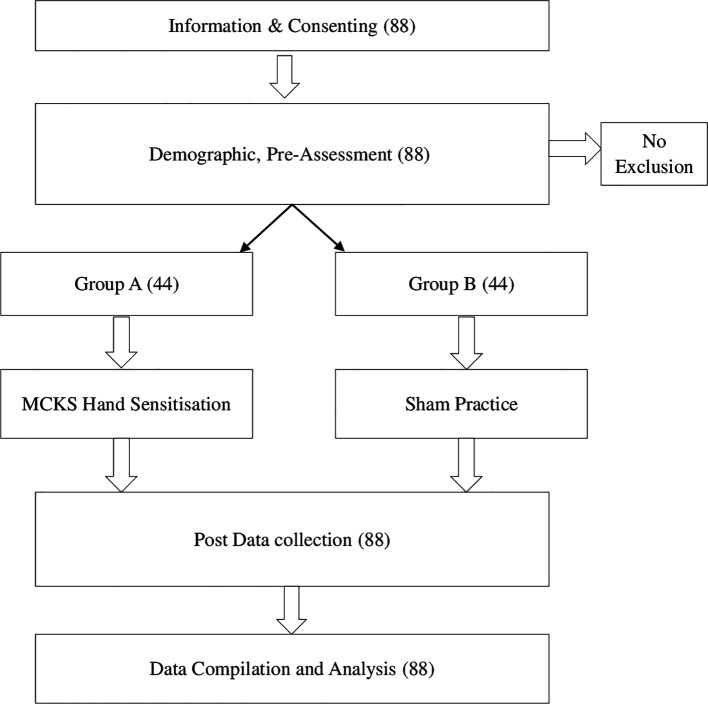
Flow chart.

Pre-service teachers who signed a consent form and expressed an interest in participating in the study were accommodated in the hall. The demographic information, learning preferences and performance test results were obtained. The participants were divided and randomised into two groups (1:1) using the fishbowl method, and the groups were assigned using the coin toss method. The study participants were blinded to their condition. To prevent interference, only one group at a time engaged in sensitization practice within the hall, while the other group spent their time in a separate hall located 200 meters away from the study hall. The entire process of data collection and procedure administration was concluded over a span of five hours ( Figure 1).

### Experimental group

Master Choa Kok Sui’s sensitization hand practise involved the steps as follows. ^7^ Practise abdominal breathing while sitting comfortably with the spine straight. Connect the tongue to the palate, press the centre of the palms with the thumb, place both palms facing each other and parallel to one another at three inches, move your hands slightly back and forth, and be aware of the centres of palms and the tips of fingers. The whole trial of Sensitization practice was conducted three times with one minute break (approximately) between each session consisting of 120 seconds.

### Sham group

The sham group got identical instructions to the experimental group, with the exception of both the palm facing in opposite directions and height variation between hands of approximately 3 inches.

### Measurements

*Demographic details*

The participants’ age, gender, locality, medications taken within the last 24 hours, and highest educational qualification were recorded. Height was measured using a measuring tape, and weight in kilograms was divided by height in meters squared to calculate the BMI (Body Mass Index) using the formula: BMI = weight (kg) /height ^2^ (m ^2^).

Self-administered questionnaires (Learning Style, Sensation of Hands timing Questionnaire) and performance tests (Six letter Cancellation Test, Ruler Drop Test) were used to measure different outcome variables.

*Honey and Mumford:* Learning Styles Questionnaire: The Learning Styles Questionnaire (LSQ) is designed to measure learning preferences in individuals aged 16+, derived from the same conceptual basis as Kolb’s Learning Style Inventory. The Learning Style Questionnaire ^10^ contains 80 statements representing four learning styles that may be predominant in an individual, the activist, the reflector, the theorist, and the pragmatist. Construct validity to Kolb’s learning styles was confirmed using factor analysis. ^28^

*Six Letter Cancellation Test:* It is a neuropsychological test that primarily measures the ability to maintain attention and concentration. A sheet of randomly printed letters of alphabet was provided with 6 target letters. Subjects can either select a single target letter at a time or all six letters at once, and can follow random, vertical, or horizontal paths on cancelling the letters. They can cancel maximum possible letters within 90 seconds of time. The assessment process included monitoring both the overall number of cancellations and the occurrences of incorrectly cancelled targeted letters. The net score was established by deducting the instances of incorrect cancellations from the total number of cancellations of targeted letters. ^29^

*Reaction Time:* To measure the Reaction time, Ruler Drop Test (RDT) was used Participants were asked to sit comfortably on a chair, ensuring that their dominant hand was positioned with the elbow bent at a 90-degree angle. The arm was then supported on the flat surface of the chair, with the open hand resting at the edge of the surface. A 60-cm-long ruler was dropped unexpectedly between the thumb and forefingers of their hand. They were instructed to quickly grasp the ruler as swiftly as possible. This ruler drop test (RDT) was repeated three times using the participant’s dominant hand. A rest interval of approximately 30 seconds was provided for each participant between each instance of dropping the ruler. The RT conversion was performed using the formula for a body in free fall under the influence of gravity (d= ½ gt ^2^). The test score is the distance reached, with a lower distance indicating better performance. ^30^

*The sensation of Hands Timing Questionnaire (SHTQ):* The Research Centre of the World Pranic Healing Foundation in India has created a new and self-administered questionnaire, which has undergone face validation. This questionnaire follows a semi-structured format and includes a combination of open-ended and closed-ended questions. These questions aim to capture information about participants’ experiences of sensations between their hands, the time it took to perceive these sensations, and how long the sensations persisted. Intense experience of sensations among hands (left, right, both) and three open-ended questions on experience between hands were involved.

The open-ended questions included were 1) How did you feel about hand sensitisation practice? 2) In your opinion, what is the reason for experiencing these sensations between your hands 3) Describe the impact of the hand sensitisation experience on you?

*Experience of sensation of hands:* To keep track of how long first experience took and how long they lasted, a stopwatch was displayed in a projector. The duration between hands that the biofield experienced throughout a 120-second period was self-reported. Experiments were repeated three times and average time has been considered. The pattern of experiences between the hands and which hand is more prominent in feeling the sensation were explored.

### Data analysis

The experiences reported by participants in each group, along with the timings recorded in the Sensation Hands Timing Questionnaire, were thematically analysed using an inductive approach that aligned with the experiences during MCKS hands sensitisation. ^8^ The meaningful words containing experiences between the hands were initially grouped by the first author. The inclusion of experiences similar to biofield sensations was then categorized by both the first and second authors. Additionally, other authors of this paper verified the data, and queries were addressed. Subject’s sensation between hands during three attempts was derived for how long it typically takes to have first experience and how long each experience lasts. The average value of sensations was calculated. Participants of time taken to first experience the sensations were categorised into no experience, 1-10 seconds, 10.1-20 seconds, 20.1-30 seconds, 30.1 to 40 seconds, and more than 40 seconds. The duration of sensation experiences was categorised into no experience, 1-20 seconds, 20.1-40 seconds, 40.1-60 seconds, 60.1-80 seconds, and more than 80 seconds. The average time for each category of biofield sensations was also calculated.

Furthermore, sensation experiences were classified as single, two sensations and three or more felt at same time. The association between the reported sensations and learning styles, as well as the association between the number of, biofield sensations and learning styles were analysed. Descriptive and inferential statistics using SPSS version 21 and Microsoft Excel were used to analyse the results.

## Results

The study comprised 88 first year students who were informed about the study and willingly participated by signing the consent form, with all participants meeting the eligibility criteria. Table 1 shows the demographic details and pre-assessment of pre-service teachers who participated in the trial. ^47^ The experimental and sham groups each consisted of 44 participants. There were 65 women (72.7% in the experimental group and 75% in the sham group). A total of 77.3% had a rural background and there was no significant difference in participants’ gender (χ ^2^ = 0.059, p = .808) or location (χ ^2^ = 1.035, p = .309) between the experimental and sham groups. The experimental group’s body mass index (BMI) was 21.95 ± 6.38, while the sham group’s BMI was 20.41 ± 3.50. No significant difference was found between the experimental and sham groups in terms of BMI (χ ^2^ = 4.286, p = .232). 14.77% of pre-service teachers had post-graduate, while the remaining 85.23% had earned their degrees prior to enrolling in the pre-service teacher training programme. Importantly, the study did not experience any losses or exclusions following randomization, and no dropouts were recorded. Based on learning style theory, very high scores were identified in Active 49 (55.68%), Theorist 48 (54.55%), Pragmatist 38 (43.1%), and Reflector learning styles 27 (30.68%) among pre-service teachers. Learning style preferences were categorized as very strong, strong, moderate, and low. No significant differences were found between the experimental and sham group pre-service teachers on active (2.295, p = .513), reflective (2.180, p = .703), theorist (1.307, p = .860), and pragmatic (3.197, p = .525) learning styles.

**Table 1.** Demographic details and pre-assessments among pre-school teachers.

Variables		Experimental		Sham		Statistic	
	Categories	N	%	N	%	χ ^2^	p- value
*Gender*	Male	12	27.3	11	25	0.059	.808
	Female	32	72.7	33	75		
*Location*	Urban	12	27.3	8	18.2	1.035	.309
	Rural	32	72.7	36	81.8		
*BMI*	Under	14	31.8	14	31.8	4.286	.232
	Normal	20	45.5	23	52.3		
	Pre-obese	6	13.6	7	15.9		
	Obese	4	9.1	0	0%		
LSP *-Active*	Very Strong	24	54.5	25	56.8	2.295	.513
	Strong	10	22.7	8	18.2		
	Moderate	10	22.7	9	20.5		
	Low	0	0	2	4.5		
LSP *-Reflective*	Very Strong	14	31.8	13	29.5	2.180	.703
	Strong	26	59.1	26	59.1		
	Moderate	3	6.8	4	9.1		
	Low	1	2.3	0	0		
	Very Low	0	0	1	2.3		
LSP *-Theorist*	Very Strong	23	52.3	24	54.5	1.307	.860
	Strong	9	20.5	9	20.5		
	Moderate	8	18.2	6	13.6		
	Low	4	9.1	4	9.1		
	Very Low	0	0	1	2.3		
LSP *-Pragmatic*	Very Strong	16	36.4	22	50.0	3.197	.525
	Strong	13	29.5	13	29.5		
	Moderate	11	25.0	5	11.4		
	Low	3	6.8	3	6.8		
	Very Low	1	2.3	1	2.3		
*Performance Tests*		Mean ± S.D.		Mean ± S.D.		t	p-value
*SLCT*		53.03 ± 15.77		47.29 ± 13.38		-1.837	p = .070
*RT (Seconds)*		0.21 ± 0.03		0.21 ± 0.03		-.104	p = .917

BMI - Body Mass Index, LSP - Learning Style Preferences, SLCT - Six Letter Cancellation Test Scores, RT - Reaction time.

Performance tests involved the Six Letter Cancellation Test (SLCT) and the Reaction Time (RT) Test. No significant differences were found between the experimental group (M = 53.03 ± 15.77) and the sham group (M = 47.29 ± 13.38) in SLCT (t = -1.837, p = .070) and RT (t = -0.104, p = .917). The mean reaction time of the experimental group and the sham group were 0.21 ± 0.0 and 0.21 ± 0.03, respectively. Right hand dominance was reported in all participants except one.

**Figure 2.**
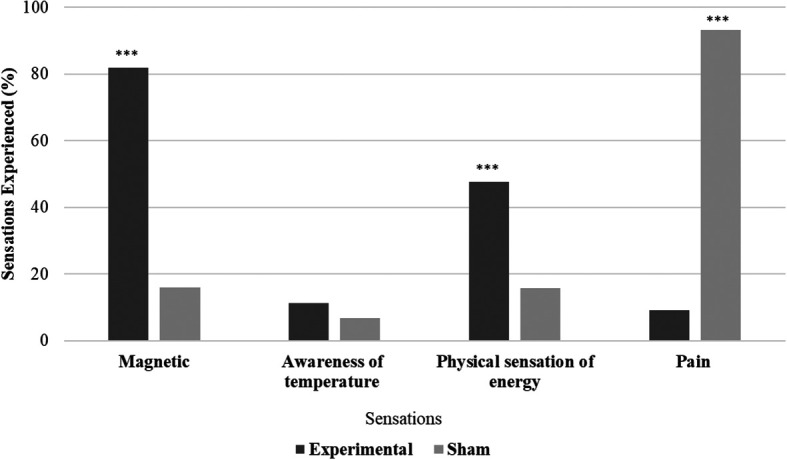
Sensations during hands sensitisation practice.

All the participants in the experimental group (N = 44) and 29.55% (N = 13) in the sham group reported experiencing biofield sensations. Significant variations in sensation experiences were observed between the experimental and sham groups (χ ^2^ = 58.47, p < .001). The experimental group had more participants reporting magnetic and physical energy sensations, while the sham group reported experiencing more pain sensations. Differences between the experimental and sham groups were examined for the presence or absence of sensations. Magnetic sensations were significantly (χ ^2^ = 38.247, p ≤ .001) higher in the experimental group (81.81%) compared to the sham group (15.91%), physical sensations of energy were also significantly (χ ^2^ = 12.02, p ≤ .001) higher in the experimental group (47.72%). Awareness of temperature sensations showed no significant difference between the groups (χ ^2^ = 0.550, p = .458), while pain sensations were significantly (χ ^2^ = 62.259, p ≤ .001) higher in the sham group (93.18%) compared to experimental group (9.09%) ( Figure 2).

The experimental group reported magnetic sensations (36/44) (“ *Magnetic pull between two palms,*” “ *magnetic attraction*”, “ *gravitational force*”, and “ *Like a magnetic connection*” etc.), (21/44) reported physical sensation of energy (“ *Felt hands are heavy*”, “ *Something circling or spinning in mid of the palms*”, “ *Felt like holding object*”, “ *Something passing between hands*”, “ *friction*”, “ *vibration*”, “ *lightness*”, “ *heaviness*”, “ *shaking*”, “ *collision, pulsating*” and “ *elastic force*” etc.), (4/44) reported pain experiences (“ *Hand pain*”, “ *shoulder pain*” and “ *pain at the tip of tongue*”) and (5/44) of them reported awareness of temperature (“ *cold*”, “ *heat*” and “ *hotness*”).

In the sham group, 93.2% (41/44) reported pain experiences (“Hand pain”, “Left hand wrist pain”, “Little pain” and “Pain and attention”), 13.6% (6/44) reported physical sensation of energy (“Felt a force like a thing between the hands”, “Hand pain with vibration”, “Weight lift feel”, “Hand pain and swimming feeling and Pulling” etc.), (7/44) reported magnetic sensations ( *“magnetic force*”, “ *Pain and magnetic*”) and (3/44) of them reported awareness of temperature (“cold” and “pain with warmness”).

Table 2 shows there was no significant difference found between category of time taken to first experience the sensation between experimental and sham group participants.

**Table 2.** Time taken to experience the sensation during MCKS hand sensitisation.

Categories		Experimental	%	Sham	%	Statistics
Time taken to first experience	No sensation	0	0	1	2.3	χ ^2^ = 5.223, p = .389
	1-10 seconds	0	0	2	4.5	
	10.1-20 seconds	4	9.1	7	15.9	
	20.1-30 seconds	10	22.7	7	15.9	
	30.1-40 seconds	9	20.5	11	25	
	More than 40 seconds	21	47.7	16	36.4	
Duration of experiences	No sensation	0	0	1	2.3	χ ^2^ = 1.167, p = .948
	1-20 seconds	7	15.9	8	18.2	
	20.1-40 seconds	10	22.7	9	20.5	
	40.1-60 seconds	11	25	10	22.7	
	60.1-80 seconds	12	27.3	12	27.3	
	More than 80 seconds	4	9.1	4	9.1	

**Figure 3.**
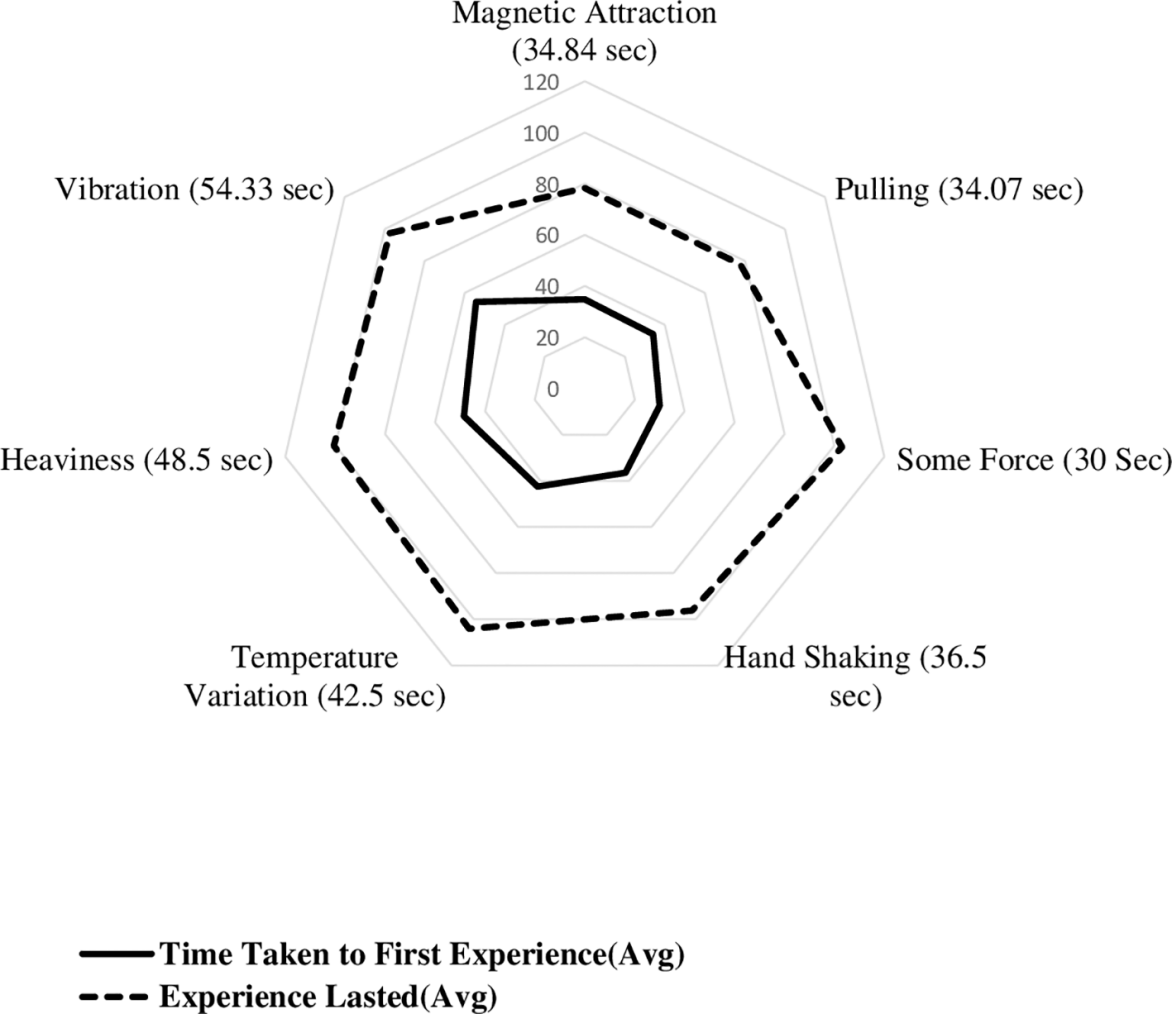
Mean time to experience different biofield sensations among experimental group participants.

The categorised experiences and mean time taken to first experience the sensations and extend of time the sensations were sustained (Duration) were identified. Figure 3 displays the radial plot on mean time to experience different biofield sensations, including the time taken to first experience various sensations and the experiences lasted. The average time it took to first experience biofield sensations of magnetic attraction, pulling sensation, some force, handshaking experience, awareness of temperature, heaviness, and vibration were 34.84, 34.07, 30, 36.50, 42.5, 48.5, and 54.33 seconds, respectively. The time it took to first experience sensations of hand shaking, lightness, pulse beating, collision, and elastic force were 37, 40, 58, 58, and 61 seconds, respectively. The shortest time it took to first experience a sensation was 5 seconds, and the duration of sensations lasted up to 120 seconds. The minimum time taken to experience magnetic, pulling, some force, hand shaking, awareness of temperature, heaviness and vibration sensations were 5, 20, 18, 22.5, 14, 32 and 26.67 seconds, respectively.

The mean duration of each sensation was calculated, with magnetic, pulling, some force, hand shaking, awareness of temperature, heaviness, and vibration having mean durations of 43.39 seconds, 43.67 seconds, 73 seconds, 59.83 seconds, 61.70 seconds, 52.00 seconds, and 43 seconds, respectively. The duration of lightness, pulse beating, collision, and elastic force were reported as single values of 80 seconds, 40 seconds, 62 seconds, and 61 seconds, respectively. Based on the thematic categorisation, mean time taken to first experience the magnetic sensation was 34.84 ± 12.97 seconds, other biofield sensations was 40.28 ± 20.96 seconds and awareness of temperature was 42.50 ± 19.79 seconds.

Participants’ reported sensations between their hands were divided into three categories: only one type of sensations (Mono), two types of sensations, and three or more sensations (Multi biofield sensations) felt at same time. More people (N = 25, 56.82%) reported having only one sort of sensation than had two types (N = 18, 40.91%), three or more sensations (N = 1, 2.27%). Table 3 shows the association of learning styles sub categories with mono and multi biofield sensations from the Sensations Hands Timing Questionnaire (SHTQ). Participants with reflective (χ ^2^ = 16.396, p = .012) and pragmatic (χ ^2^ = 16.653, p = .034) learning styles sub categories had a significant association with mono and multi biofield sensation experiences between hands. Additionally, 34.09% of Strong reflective and 20.45% with very high pragmatic learning were more likely to experience mono sensations than multi sensations.

**Table 3.** Association of mono and multi biofield sensations and learning style preferences among experimental group participants.

Variables		Mono and multi biofield sensations								Statistics
Learning Styles	Categories	Single	%	Two	%	Three or more	%	Total	%	χ ^2^ p -value
**Active**	*Very strong*	12	27.27	11	25	1	2.27	24	54.54	1.789, .775
	*Strong*	6	13.64	4	9.0	0	0	10	22.73	
	*Moderate*	7	15.91	3	6.82	0	0	10	22.73	
**Reflective**	*Very Strong*	8	18.18	6	13.64	0	0	14	31.82	16.396, .012
	*Strong*	15	34.09	11	25	0	0	26	59.09	
	*Moderate*	2	4.55	0	0	1	2.27	3	6.82	
	*Low*	0	0	1	2.27	0	0	1	2.27	
**Theoretic**	*Very Strong*	12	27.27	11	25	0	0	23	52.27	12.390, .054
	*Strong*	6	13.64	3	6.82	0	0	9	20.45	
	*Moderate*	6	13.64	2	4.55	0	0	8	18.18	
	*Low*	1	2.27	2	4.55	1	2.27	4	9.09	
**Pragmatic**	*Very Strong*	9	20.45	7	15.91	0	0	16	36.36	16.653, .034
	*Strong*	6	13.64	7	15.91	0	0	13	29.55	
	*Moderate*	7	15.91	4	9.09	0	0	11	25	
	*Low*	2	4.55	0	0	1	2.27	3	6.82	
	*Very Low*	1	2.27	0	0	0	0	1	2.27	

**Figure 4.**
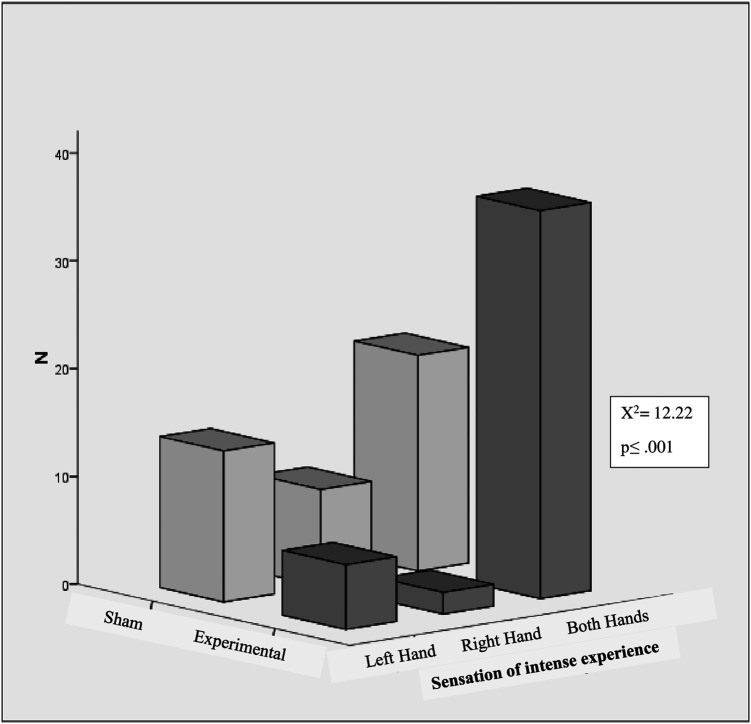
Intense experience of sensations in hands.

Figure 4 shows the intense experience of sensation in hands. A significant difference was found between the experimental and sham groups in the intense experience of sensation in the hands (left, right, and both) (χ ^2^ = 12.22, p < .001, N = 87). The intense experience of sensations in hands within the experimental group also were differed depending on whether they occurred in the left hand, right hand, or both hands (χ ^2^ = 47.09, p < .001, N = 44). In the experimental group, 81.81% (36) reported the intensity of sensation experiences in both hands. 13.6% (6/44) of participants reported experiencing intensity at right hand, while only 4.54% (2/44) reported experiencing sensations in their left hand. In the sham group, 35.7% of participants experienced the intensity of the sensation in both hands, while 20.9% and 32.6% experienced the intensity in their left and right hands, respectively. However, within the sham group, the intensity of sensations experienced did not differ significantly between the left hand, right hand, or both hands.

A significant negative correlation (r = -0.240, p = .025) exists between the time taken to first experience sensation and duration of experiences among all participants. In the experimental group, the duration of experiences and the time taken to first experience a sensation were negatively correlated (r = -0.326, p = .009). No other correlations were found to be significant.

**Figure 5.**
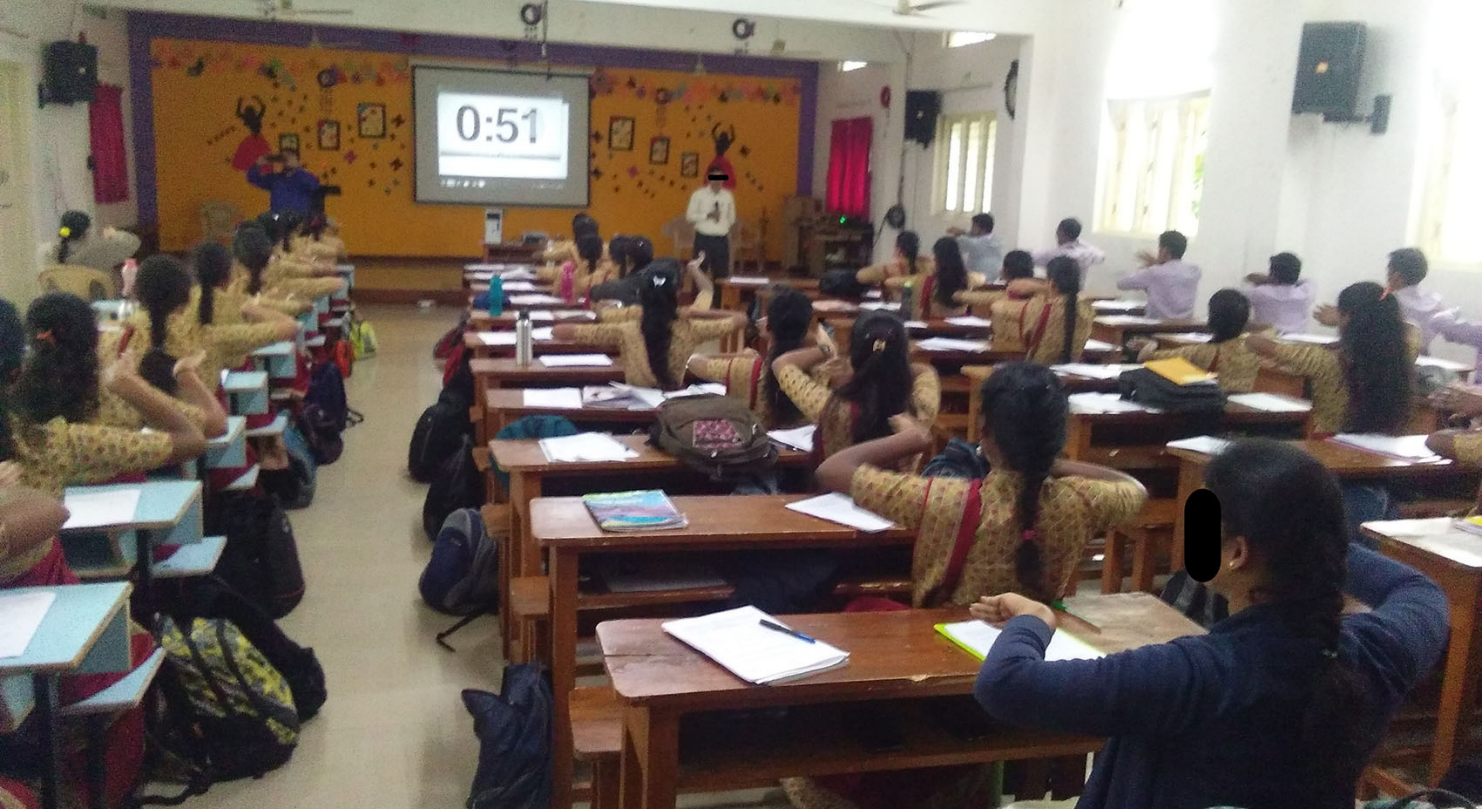
Experimental sessions of Sham group. All participants gave consent for their picture to be used.

In the open-ended questions, participants expressed that the experiences were magical, good, and novel sensations were felt during the practice. Improvement in respiration, reduction of stress, calmness and improvement in mood and concentration were common among experimental group participants as shown in Table 4. Participants reported their opinion about the reason for experiencing biofield sensations and the impact of hands sensitisation practice. The reasons participants reported for experiencing the sensations included attention and concentration, blood circulation, sense of calm and the coordination of sensory organs in the body, among others. Self-reported and sparsely described feedback was noticed in sham group ( Figure 5).

**Table 4.** Qualitative responses to open-ended questions.

Experimental group	Sham group
**In your opinion, what is the reason for experiencing these sensations between your hands?**	
*“May be because of attentively concentrated. There might be some inter relation between hands” - **E3***	“ *Nothing significant” - **P4***
*“Our body contains so many ionic particles, when we rub the hands and relax and moving, the ionic particle attracts other particles” - **E6***	“ *Right hand pain” - **P6***
*“Five sensory organs coordinate with one another like tongue touches upper nasal region and all concentration lay on movement of hand and respiration” - **E9***	“ *I do not feel anything” - **P24***
*“I think circulation of blood and movement of oxygen throughout the hands/body” - **E18***	“No experience *” - **P10***
*“According to me, my curiosity, attention and concentration and my involvement is the reason” - **E29***	*“Opposite direction and magnetic force” - **P32***
**Describe the impact of the hand sensitization experience on you?**	
*“Freeness to hands and it helps to control respiration rate by ourselves” - **E9***	*“Improvement in the breathing and concentration” - **P34***
*“I think I feel cool mind. It is like a one type exercise for me. My stress and hands become free” - **E8***	*“It was a different experiment and would like to try it again” - **P28***
*“I feel, I am released of mental stress, my mind is fee and felt like a load of tension is removed from my mind” - **E16***	“ *Got awareness regarding hand movement and its reaction” - **P17***
*“I have a sense of calm. I have more anger and less patience. But from this experiment, I found that patience, calmness is a medicine for anger. Not having habit of meditation but it helped me” - **E37***	*“No impact on me - **P10***
*“If we do it daily, there will be no thoughts. Our mood will be good. It helps to learning” - **E30***	*“I felt hand power increased” - **P9***

## Discussion

This randomized controlled trial on sensitization between hands has demonstrated that both the experimental and sham groups self-reported an experience biofield sensations ( *prana*) in between their hands. Furthermore, it has been determined that the interpretation of these sensations based on time is a viable approach. The palm area of the human body exhibits greater sensitivity and a higher ability to discriminate tactile stimuli compared to other areas of the skin, as evidenced by a survey of 18 people found that they perceived spontaneous sensations was more in their hands than feet. ^31^ In the current study, 57 participants experienced biofield sensation between their hands. The sham group participants experienced less (13) biofield sensations compared to experimental group (44). This significant disparity suggests that the sensitisation between hands used in the experimental group had a tangible impact on sensory perception, beyond the placebo effect. The practice of sensitizing the hands primarily focuses on the minor *chakras* located in the centre of the palms and mini chakras in each finger tips have the capability of absorbing and projecting prana. Furthermore, positioning the palms facing each other enhance perceptual abilities in sensing *prana*, possibly due to the close interaction of energy points in both hands. ^7^

Majority of sham group participants reported hand, wrist, and arm pain during the experiment. It is possible that during the experience of pain, individuals may unconsciously direct their attention towards the affected area in the hands. ^32^ The likelihood of not experiencing sensations could be due to the hand sensitizing procedure and the hand's sensitivity level. This may be one of the reasons for not experiencing biofield sensations. Regarding pain in the sham group, it may relate to the positioning of their palms and hands. Hence the data on the chronometry and biofield energy experiences of the experimental group were analysed further based on duration. The time taken to first experience the sensations of magnetic, other biofield sensations and awareness of temperature are 34.84 seconds, 40.28 seconds and 42.50 seconds, respectively.

People who frequently experience spontaneous sensations in their left hand, do so because of the dominant activity of the right hemisphere of the brain. ^23^ In this investigation, biofield sensations were experienced in both hands by the participants, who reported the intensity of the experiences in both hands. It is possible that both hemispheres were activated during MCKS hands sensitisation. Those who followed MCKS Hands sensitisation procedure felt different biofield sensations in between hands, which positively affected the physical and psychological wellbeing. ^33^ Biofield sensations have been reported following specific guidance or procedures mentioned in biofield practices. ^34^

Somatic spontaneous sensations such as tingling, temperature, beats, pulses, and pins have been previously investigated by detecting these sensations within 10 seconds. Older participants reported more spontaneous sensations and longer duration of sensations compared to the youth population. ^35^ Magnetic field, self-awareness, sensitivity to others and conscious intent are the factors influencing biofield awareness. ^16^^,^^36^^–^^39^ Therefore, the chances of variations in sensations are natural. MCKS sensitisation has been used as a learning practice to explore about the subtle sensations or *pranic energy* sensations. ^7^ According to the findings, different participants experienced biofield sensations for varying lengths of time during hand sensitization practice. In depth research into how these factors influence the time it takes to experience biofield sensations could lead to a more precise understanding of the mechanism at play.

This study explored a cognitive factor related to learning style preferences and its association with biofield sensations among pre-service teachers. Based on the context, environment, teaching method and subject of learning, the learning style preferences may get changed. ^40^ There was no association found between sensation between hands and learning styles in this study, but the learning styles were associated to number of biofield sensations felt during sensitising the hands. According to the attention processing model, participants are able to pay attention to a specific experience due to the difficulty of observing all other sensations at the same time. ^41^ Self-analysis or introspection is at the core of reflective learning. Complex Judgemental skills and problem-solving abilities are essential qualities of reflective thinkers. ^42^ Recording the intricate interplay of various sensations over time can be challenging for self-interpretation. Consequently, participants might have opted to focus on individual biofield experiences throughout the entire practice session. For instance, sensations like “Magnetic with Cold or Warm” or “Pulling along with Other Sensations,” or experiencing three or more sensations simultaneously, could have posed difficulties in accurately reporting their timings. Similarly, individuals who lean toward pragmatic approaches tend to simplify matters by highlighting single experiences when drawing conclusions. Hence, participants with higher reflective and pragmatic tendencies displayed a tendency to articulate individual sensations rather than complex combinations as their reported experiences.

The negative correlation between the time taken to have the first experience and the overall duration of experiences in the experimental group suggests a complex relationship influenced by factors such as learning, adaptation, and familiarity.The time taken to experience biofield sensation were unrelated to reaction time, sustained attention, or body mass index. Research has found that, paying attention to the hands improves the duration of experiences. ^35^ Separating the conscious and unconscious sensory processes and connecting with attention is a difficult task. ^43^ Mind wandering awareness has been linked to perception of spontaneous stimulus. ^44^ Evidence suggests that the intensity of experience influences body mass index. ^45^ Participants can gain a better understanding of biofield experiences by receiving further training and learning about biofield-related therapy. ^46^ In this study, hands-sensitization practise in the experimental group had a favourable effect on the participants, and qualitative feedback supported the idea that biofield sensations improve current moods or states. ^33^

The replicability of this procedure offers potential for future biofield research, with follow-up studies involving diverse participant groups to validate the consistency of observed results related to the time taken to experience biofield sensations.

### Limitations of this study

The study’s questionnaire is unstandardized, necessitating the need to develop an instrument to measure time-related sensations between hands. Participants aged 18-24 were included, excluding older populations.

### Implications

This study findings may inspire biofield researchers to conduct time-based cognition and perception experiments. Based on MCKS hand sensitization, a theory on biofield sensations might be constructed, which could then be utilised as a tool or technique for studying numerous subtle sensations.

### Future direction

Further research on the duration of biofield sensations using chronometry is needed. Research is needed on specific biofield sensation-based approaches, as neuropsychological evidence on palm sensations is limited. It’s unclear if cortical brain activation is related to pranic energy experiences. Both spontaneous sensations and MCKS Hands Sensitisation experiences start without stimulus, but differ in methods. Investigation is needed to understand similarities and differences.

## Conclusion

This study demonstrated that hand sensitisation effectively enhanced biofield sensory experiences, with the experimental group reporting significantly more sensations compared to the sham group.

This research emphasises the significance of recognising biofield sensations in hands sensitization practises. The MCKS hands sensitization practise research looked at biofield sensations between the hands under controlled settings. It kept track of time-related evaluations. It took 34.84 seconds, 54.33 seconds and 42.5 seconds on average to initially feel magnetic attraction, vibration and awareness of temperature between hands. A notable finding was the negative correlation observed between the time taken to first experience a sensation and the overall duration of these experiences, suggesting a complex relationship influenced by factors such as learning and adaptation. The study highlights the role of cognitive processes in biofield perception and supports the potential for further research to explore these mechanisms and validate the consistency of these effects across diverse groups.

## Data Availability

Figshare: Time to experience Biofield Sensations Between Hands – A Randomized Control Trial, https://doi.org/10.6084/m9.figshare.24024948.v1. ^47^ This project contains the following underlying data:
- Consent and Demographic Details.pdf - Master Sheet_ Pre service Teachers.xlsx Figshare: CONSORT checklist for ‘Time to sense biofield (Prana) experiences between hands: A preliminary single blinded randomized controlled trial’, https://doi.org/10.6084/m9.figshare.24024948.v1. ^47^ Data are available under the terms of the Creative Commons Attribution 4.0 International license (CC-BY 4.0).
