# Supplementary material for: Time to sense biofield (Prana) experiences between hands: A preliminary single blinded randomized placebo controlled trial
Source: F1000Res. 2024 Aug 20;12:1536. Originally published 2023 Nov 30. [Version 5] doi: 10.12688/f1000research.139737.5 (PMC11208854; doi:10.12688/f1000research.139737.5)
Supplement: Peer review history

# Reviewer report: Reviewer response for version 5

**DOI:** 10.5256/f1000research.170299.r309960

**Published:** 2024-08-24

**Authors:** Sudhanshu Kacker

**Affiliations:** 1 Rajasthan University of Health Sciences, Jaipur,, Rajasthan,, India

I have read all modifications and approved this revised manuscript.

 Congratulations. Hard work is appreciable.

Is the work clearly and accurately presented and does it cite the current literature?

Partly

If applicable, is the statistical analysis and its interpretation appropriate?

Partly

Are all the source data underlying the results available to ensure full reproducibility?

Yes

Is the study design appropriate and is the work technically sound?

Yes

Are the conclusions drawn adequately supported by the results?

Partly

Are sufficient details of methods and analysis provided to allow replication by others?

Partly

Reviewer Expertise:

Physiology

I confirm that I have read this submission and believe that I have an appropriate level of expertise to confirm that it is of an acceptable scientific standard.

# Reviewer report: Reviewer response for version 5

**DOI:** 10.5256/f1000research.170299.r315468

**Published:** 2024-08-24

**Authors:** Ann Linda Baldwin

**Affiliations:** 1 The University of Arizona, Tucson, Arizona, USA

The response to my critique regarding legend for figure 4 is appropriate except in the first sentence: "Figure 4 shows the numbers of participants who detected an intense experience of sensation in there left, right or both hands". "there" should be changed to "their".

Is the work clearly and accurately presented and does it cite the current literature?

Partly

If applicable, is the statistical analysis and its interpretation appropriate?

Yes

Are all the source data underlying the results available to ensure full reproducibility?

Yes

Is the study design appropriate and is the work technically sound?

Yes

Are the conclusions drawn adequately supported by the results?

Partly

Are sufficient details of methods and analysis provided to allow replication by others?

Partly

Reviewer Expertise:

Physiology of mind-body interactions

I confirm that I have read this submission and believe that I have an appropriate level of expertise to confirm that it is of an acceptable scientific standard.

# Reviewer report: Reviewer response for version 4

**DOI:** 10.5256/f1000research.169791.r309959

**Published:** 2024-08-06

**Authors:** Ann Linda Baldwin

**Affiliations:** 1 The University of Arizona, Tucson, Arizona, USA

The authors have not responded to my comments on the last version of their manuscript in any way. The text for Figure 4 has not been changed. I do not approve of this version of the manuscript.

Is the work clearly and accurately presented and does it cite the current literature?

Partly

If applicable, is the statistical analysis and its interpretation appropriate?

Yes

Are all the source data underlying the results available to ensure full reproducibility?

Yes

Is the study design appropriate and is the work technically sound?

Yes

Are the conclusions drawn adequately supported by the results?

Partly

Are sufficient details of methods and analysis provided to allow replication by others?

Partly

Reviewer Expertise:

Physiology of mind-body interactions

I confirm that I have read this submission and believe that I have an appropriate level of expertise to confirm that it is of an acceptable scientific standard, however I have significant reservations, as outlined above.

# Response

**Published:** 2024-08-12

**Authors:** Srikanth Jois

**Affiliations:** Research Centre, World Pranic Healing Foundation India, Mysuru, Karnataka, India

**Reviewer Query:** The authors have not responded to my comments on the last version of their manuscript in any way. The text for Figure 4 has not been changed. I do not approve of this version of the manuscript.

**Author Response:** We apologize for overlooking the comments made by the Reviewer. We have now incorporated the Reviewer's suggestions into the text of Figure 4, as recommended.

# Reviewer report: Reviewer response for version 3

**DOI:** 10.5256/f1000research.169098.r306546

**Published:** 2024-07-29

**Authors:** Sudhanshu Kacker

**Affiliations:** 1 Rajasthan University of Health Sciences, Jaipur,, Rajasthan,, India

The topic of the manuscript is very interesting and required, which I have mentioned in previous  communication as well

 Thanks to the author for all the work put into the manuscript. It is improved from its original versions. Some minor changes to address include the following:

 1. Add details of sociodemographic information.

 2. Study flow chart should be revised.

 3. In conclusion, results  findings (numerical values) should be deleted.

Is the work clearly and accurately presented and does it cite the current literature?

Partly

If applicable, is the statistical analysis and its interpretation appropriate?

Partly

Are all the source data underlying the results available to ensure full reproducibility?

Yes

Is the study design appropriate and is the work technically sound?

Yes

Are the conclusions drawn adequately supported by the results?

Partly

Are sufficient details of methods and analysis provided to allow replication by others?

Partly

Reviewer Expertise:

Noncommunicable diseases ,reaction time, physical fitness, life style modifications

I confirm that I have read this submission and believe that I have an appropriate level of expertise to confirm that it is of an acceptable scientific standard.

# Response

**Published:** 2024-07-29

**Authors:** Srikanth Jois

**Affiliations:** Research Centre, World Pranic Healing Foundation India, Mysuru, Karnataka, India

**Query**

**Response**

**Location of Changes Implemented**

**Sudhanshu Kacker, **Rajasthan University of Health Sciences, Jaipur, Rajasthan, India

 Query: Add details of sociodemographic information.

 Response: Sociodemographic details are already provided in the paper. Table 1 and in the Results section 

 Query: Study flow chart should be revised.

 Response: It is revised as suggested in Figure 1

 Query: In conclusion, results findings (numerical values) should be deleted.

 Response: Since the numerical values are important and signifies the outcome of the entire study it is retained. It will be easy for readers to understand the average time to sense or feel biofield sensations between hands while practicing hands sensitization technique.

# Reviewer report: Reviewer response for version 3

**DOI:** 10.5256/f1000research.169098.r306548

**Published:** 2024-07-25

**Authors:** Saamdu Chetri

**Affiliations:** 1 Shoolini University, Solan, Himachal Pradesh, India

Approved!

Is the work clearly and accurately presented and does it cite the current literature?

Yes

If applicable, is the statistical analysis and its interpretation appropriate?

Yes

Are all the source data underlying the results available to ensure full reproducibility?

Partly

Is the study design appropriate and is the work technically sound?

Yes

Are the conclusions drawn adequately supported by the results?

Yes

Are sufficient details of methods and analysis provided to allow replication by others?

Yes

Reviewer Expertise:

The study can be bettered; see my suggestions.

I confirm that I have read this submission and believe that I have an appropriate level of expertise to confirm that it is of an acceptable scientific standard.

# Reviewer report: Reviewer response for version 3

**DOI:** 10.5256/f1000research.169098.r306547

**Published:** 2024-07-25

**Authors:** Ann Linda Baldwin

**Affiliations:** 1 The University of Arizona, Tucson, Arizona, USA

Please correct new text describing Figure 4 as follows (corrections shown in quotations):

 Figure 4 shows the "numbers of participants who detected an ****intense experience of sensation in their left, right or both hands".  A significant difference was found between the experimental and sham groups in the intense experience of sensation in the hands (left, right, and both) (χ ^2^ = 12.22, p < .001, N = 87). "The intense experience of sensations in hands within the experimental group also (deleted "were") differed depending on whether they occurred in the left hand, right hand, or both hands (χ ^2^ = 47.09, p < .001, N = 44)". "In the experimental group, 81.81% (36) reported the intensity of sensation experiences in both hands. 13.6% (6/44) of participants reported experiencing intensity in their right hand, while only 4.54% (2/44) reported experiencing sensations in their left hand.”

Is the work clearly and accurately presented and does it cite the current literature?

Partly

If applicable, is the statistical analysis and its interpretation appropriate?

Yes

Are all the source data underlying the results available to ensure full reproducibility?

Yes

Is the study design appropriate and is the work technically sound?

Yes

Are the conclusions drawn adequately supported by the results?

Partly

Are sufficient details of methods and analysis provided to allow replication by others?

Partly

Reviewer Expertise:

Physiology of mind-body interactions

I confirm that I have read this submission and believe that I have an appropriate level of expertise to confirm that it is of an acceptable scientific standard.

# Reviewer report: Reviewer response for version 2

**DOI:** 10.5256/f1000research.167192.r288195

**Published:** 2024-06-26

**Authors:** Saamdu Chetri

**Affiliations:** 1 Shoolini University, Solan, Himachal Pradesh, India

Some suggestions and feedback:

1. It is not very clear from the article if both hands were used for biofield experiments. Sensation between the two hands have been used. It would be helpful if biofield of the non-dominant hand was also done.
2. The study could have an intervention for the non-dominant hand to find how it can be dominant and how if both hands became dominant, what would change as a neural activity in human beings. 
3. What could be the reasons for the difference between the experimental and sham groups in sensation experienced and why. This needs more deliberation. 
4. Table 2 needs separation between the categories.
5. Can the reflex of the dominant hand change with hand rubbing and biofield? This was not clear.
6. Fig 2 indicated there is a difference between sensation expressed between the groups, while in Fig 4, perceived intensity is higher in sham group excepting both hands - it must come out with clear explanation, why?
7. Under the discussion, two points - one palm and fingers according to acupressure and anatomy has numerous connections to different sense organs of the body. Two, if one directs the attention to the affected areas, according to mindfulness, the pain should be normalized rather than increased. There must be other reason why sham group had high pain intensity. 

Is the work clearly and accurately presented and does it cite the current literature?

Yes

If applicable, is the statistical analysis and its interpretation appropriate?

Yes

Are all the source data underlying the results available to ensure full reproducibility?

Partly

Is the study design appropriate and is the work technically sound?

Yes

Are the conclusions drawn adequately supported by the results?

Yes

Are sufficient details of methods and analysis provided to allow replication by others?

Yes

Reviewer Expertise:

The study can be bettered; see my suggestions.

I confirm that I have read this submission and believe that I have an appropriate level of expertise to confirm that it is of an acceptable scientific standard.

# Response

**Published:** 2024-07-12

**Authors:** Srikanth Jois

**Affiliations:** Research Centre, World Pranic Healing Foundation India, Mysuru, Karnataka, India

**Reviewer3, Saamdu Chetri**

- It is not very clear from the article if both hands were used for biofield experiments. Sensation between the two hands have been used. It would be helpful if biofield of the non-dominant hand was also done.

Response: It is provided

 Methods-Experimental group

- The study could have an intervention for the non-dominant hand to find how it can be dominant and how if both hands became dominant, what would change as a neural activity in human beings. 

Response: In this study since both hands were used, we are unsure about the non-dominant hand. Also, most of the participants were right hand dominant and this is mentioned in the paper

 Reported in the results of Reaction time

- What could be the reasons for the difference between the experimental and sham groups in sensation experienced and why. This needs more deliberation. 

Response: It has been mentioned in discussion

 Discussion

- Table 2 needs separation between the categories.

Response: It is done

 Table 2

- Can the reflex of the dominant hand change with hand rubbing and biofield? This was not clear

Response: In this study, participants were asked to press the center of the palm for both the groups and this is mentioned in the method. Rubbing is not done in this study.  

 Method – Experimental group and Sham group

- Fig 2 indicated there is a difference between sensation expressed between the groups, while in Fig 4, perceived intensity is higher in sham group excepting both hands - it must come out with clear explanation, why?

Response: In the sham group, the intense experience included pain also and it was higher in right and left hand, than the experimental group.

 Figure 4

- Under the discussion, two points - one palm and fingers according to acupressure and anatomy has numerous connections to different sense organs of the body. Two, if one directs the attention to the affected areas, according to mindfulness, the pain should be normalized rather than increased. There must be other reason why sham group had high pain intensity. 

Response: It is provided in the discussion section

# Reviewer report: Reviewer response for version 2

**DOI:** 10.5256/f1000research.167192.r288197

**Published:** 2024-06-24

**Authors:** Sudhanshu Kacker

**Affiliations:** 1 Rajasthan University of Health Sciences, Jaipur,, Rajasthan,, India

- Overall, the introduction is not influential. This portion needs to be rewritten. A good way is to start with a proper background (introduction to the topic), describe the complementary therapies, highlight the gap area, and then link it with biofield sensation. At the end of the study, add novelty and need for the study
- Methodology: needs more details about randomized controlled participant blinded design, groups division, rationale about the age group, how subjects was screened and recruited, details of demographic variables, inclusion. exclusion criteria should be revised exclude neuromuscular disorders patients, kyphosis, scoliosis mental disorder patients, Validation of semi structured questionnaire requires adding details of statistical analysis tools, details of appropriate test, 
- Results **:** Results are poorly written. Corrections, rephrasing, explanation, and modification are needed. In demographic information, education level and socioeconomic status should be included 
- The discussion is poorly written. A lot of grammatical errors and poorly structured statements are found. There is no logical sequence of ideas.
- It is presumed that the author(s) in the discussion wanted to provide the data from the previous research. If this is true, then please discuss your results clearly and link to the earlier findings.
- The author rewrites the discussion with studies based on context and environment. teaching method and subject of learning
- The information presented in discussion section doesn’t have any link to each other.
- Limitations and Conclusion Section revised.
- Mention  limitations of study and sources of potential bias 
- Author should add further extension of study on large scale
- Conclusion: Please re elaborate conclusion based on result of study
- No hypothetical facts

Is the work clearly and accurately presented and does it cite the current literature?

Partly

If applicable, is the statistical analysis and its interpretation appropriate?

Partly

Are all the source data underlying the results available to ensure full reproducibility?

Yes

Is the study design appropriate and is the work technically sound?

Yes

Are the conclusions drawn adequately supported by the results?

Partly

Are sufficient details of methods and analysis provided to allow replication by others?

Partly

Reviewer Expertise:

Noncommunicable diseases ,reaction time, physical fitness, life style modifications

I confirm that I have read this submission and believe that I have an appropriate level of expertise to confirm that it is of an acceptable scientific standard, however I have significant reservations, as outlined above.

# Response

**Published:** 2024-07-12

**Authors:** Srikanth Jois

**Affiliations:** Research Centre, World Pranic Healing Foundation India, Mysuru, Karnataka, India

**Location of Changes Implemented**

**Sudhanshu Kacker, **Rajasthan University of Health Sciences, Jaipur, Rajasthan, India

 Introduction:

 Overall, the introduction is not influential. This portion needs to be rewritten. A good way is to start with a proper background (introduction to the topic), describe the complementary therapies, highlight the gap area, and then link it with biofield sensation. At the end of the study, add novelty and need for the study

- Thank you for your feedback. It is now addressed as suggested
- Introduction
- Methodology:

needs more details about randomized controlled participant blinded design, groups division

- Methodology is described in detail including randomization, blinding and group division
- Methods – study design

Rationale about the age group

- Rationale is provided
- Methods – study design

How subjects was screened and recruited

- It is provided
- Methods – study design

details of demographic variables

- It is provided and in table 1
- Results and Table 1

Exclusion criteria should be revised exclude neuromuscular disorders patients, kyphosis, scoliosis mental disorder patients

- Thank you for your suggestion. We will consider during our future trials.Our focus was on relatively healthy students for this study, and the current exclusion criteria cover the main health concerns that could impact the study’s outcomes. The medication details were also collected through nurse.
- Study design

Validation of semi structured questionnaire requires adding details of statistical analysis tools, details of appropriate test

- It is a face validated scale only, and is mentioned as study limitation.

Measurements -

- *The sensation of Hands Timing Questionnaire (SHTQ) and in *Limitations

The discussion is poorly written. A lot of grammatical errors and poorly structured statements are found. There is no logical sequence of ideas.

- It has been now revised
- Discussion

The information presented in discussion section doesn’t have any link to each other.

- Discussion is revised
- Discussion

Mention limitations of study and sources of potential bias 

- Limitations are provided. Bias aspect is none to declare in this paper. Discussion 4 ^th^ para and future direction.

Please re elaborate conclusion based on result of study

- It is now elaborated
- Conclusion

# Reviewer report: Reviewer response for version 2

**DOI:** 10.5256/f1000research.167192.r287322

**Published:** 2024-06-11

**Authors:** Ann Linda Baldwin

**Affiliations:** 1 The University of Arizona, Tucson, Arizona, USA

The revised version of this manuscript is far superior to the original. The protocol is better explained and the new figures provide a much clearer representation of the data than the original ones.

 The authors have satisfactorily answered my questions.

Is the work clearly and accurately presented and does it cite the current literature?

Partly

If applicable, is the statistical analysis and its interpretation appropriate?

Yes

Are all the source data underlying the results available to ensure full reproducibility?

Yes

Is the study design appropriate and is the work technically sound?

Yes

Are the conclusions drawn adequately supported by the results?

Partly

Are sufficient details of methods and analysis provided to allow replication by others?

Partly

Reviewer Expertise:

Physiology of mind-body interactions

I confirm that I have read this submission and believe that I have an appropriate level of expertise to confirm that it is of an acceptable scientific standard.

# Reviewer report: Reviewer response for version 1

**DOI:** 10.5256/f1000research.153036.r265638

**Published:** 2024-05-15

**Authors:** Ann Linda Baldwin

**Affiliations:** 1 The University of Arizona, Tucson, Arizona, USA

General Statements

 This study addresses an interesting topic, exploration of sensing the biofield between the hands, focusing on the time taken to sense the field. The number of participants included is statistically robust, and they are randomly assigned to an experimental or control group. The protocol is well described and most of the measurement tools are tested and well established. The analytical procedure appears to be adequate. However, there are problems with lack of clarity in the presentation of results and with the interpretation of observed differences in the results between the experimental and control groups.

 Major Criticisms

 Abstract

1. The terms “Ruler Drop Test and Six Letter Cancellation Test are used without explanation of what they measure.
2. It is stated that there is a significant difference in sensations of temperature variation between the groups, but this is not consistent with Figure 2.
3. Units of time (seconds?) are missing
4. There is no proper conclusion. Saying that something “was described” is not a conclusion.

Methods

 Measurements. The Six Letter Cancellation Test is badly described and there is no explanation of what it is supposed to measure.

 Results

1. A main result is that a much larger percentage of the control group experienced pain than the experimental group. To this reviewer, the obvious reason for this difference is that holding ones palms facing away from each other is much more difficult and uncomfortable than holding ones palms facing each other, and so could cause pain but this possibility is not even mentioned.
2. Figure 2 only shows one generalized statistic comparing experimental and control groups. Please supply statistics for each of the different types of sensations.
3. Are the data from Figure 3 and Table 3 from ALL participants or just the experimental group?
4. Table 3 is difficult to understand. There are too many comparisons. What parameters are the listed p-values comparing?

Discussion

 In paragraph one, discuss reasons for why the experimental group participants felt more pain than the control group (such as the reason suggested by this reviewer). What is the point of comparing sensations in palms facing versus palms directed away? This was never explained. Maybe a more interesting comparison could be both groups with palms facing but only one performing abdominal breathing

 Minor Criticisms

 Results: Define SHTQ

 Comments to Editor

 Although this study has many strengths, the major flaws concerning data presentation and interpretation need to be addressed.

Is the work clearly and accurately presented and does it cite the current literature?

Partly

If applicable, is the statistical analysis and its interpretation appropriate?

Yes

Are all the source data underlying the results available to ensure full reproducibility?

Yes

Is the study design appropriate and is the work technically sound?

Yes

Are the conclusions drawn adequately supported by the results?

Partly

Are sufficient details of methods and analysis provided to allow replication by others?

Partly

Reviewer Expertise:

Physiology of mind-body interactions

I confirm that I have read this submission and believe that I have an appropriate level of expertise to confirm that it is of an acceptable scientific standard, however I have significant reservations, as outlined above.
